# Prediction of chemical warfare agents based on cholinergic array type meta-predictors

**DOI:** 10.1038/s41598-022-21150-2

**Published:** 2022-10-06

**Authors:** Surendra Kumar, Chandni Kumari, Sangjin Ahn, Hyoungrae Kim, Mi-hyun Kim

**Affiliations:** 1 Department of Pharmacy, Gachon Institute of Pharmaceutical Science, College of Pharmacy, Gachon University, 191 Hambakmoeiro, Yeonsu-gu, Incheon, Republic of Korea; 2 Department of Artificial Intelligence, Ajou University, Suwon, 16499 Republic of Korea; 3 Department of Data Management, KEIS, 56 Mullae-ro 20-gil, Yeongdeungpo-gu, Seoul, Republic of Korea

**Keywords:** Computational biology and bioinformatics, Neuroscience, Natural hazards, Risk factors

## Abstract

Molecular insights into chemical safety are very important for sustainable development as well as risk assessment. This study considers how to manage future upcoming harmful agents, especially potentially cholinergic chemical warfare agents (CWAs). For this purpose, the structures of known cholinergic agents were encoded by molecular descriptors. And then each drug target interaction (DTI) was learned from the encoded structures and their cholinergic activities to build DTI classification models for five cholinergic targets with reliable statistical validation (ensemble-AUC: up to 0.790, MCC: up to 0.991, accuracy: up to 0.995). The collected classifiers were transformed into 2D or 3D array type meta-predictors for multi-task: (1) cholinergic prediction and (2) CWA detection. The detection ability of the array classifiers was verified under the imbalanced dataset between CWAs and none CWAs (area under the precision-recall curve: up to 0.997, MCC: up to 0.638, F1-score of none CWAs: up to 0.991, F1-score of CWAs: up to 0.585).

## Introduction

**Figure 1:**
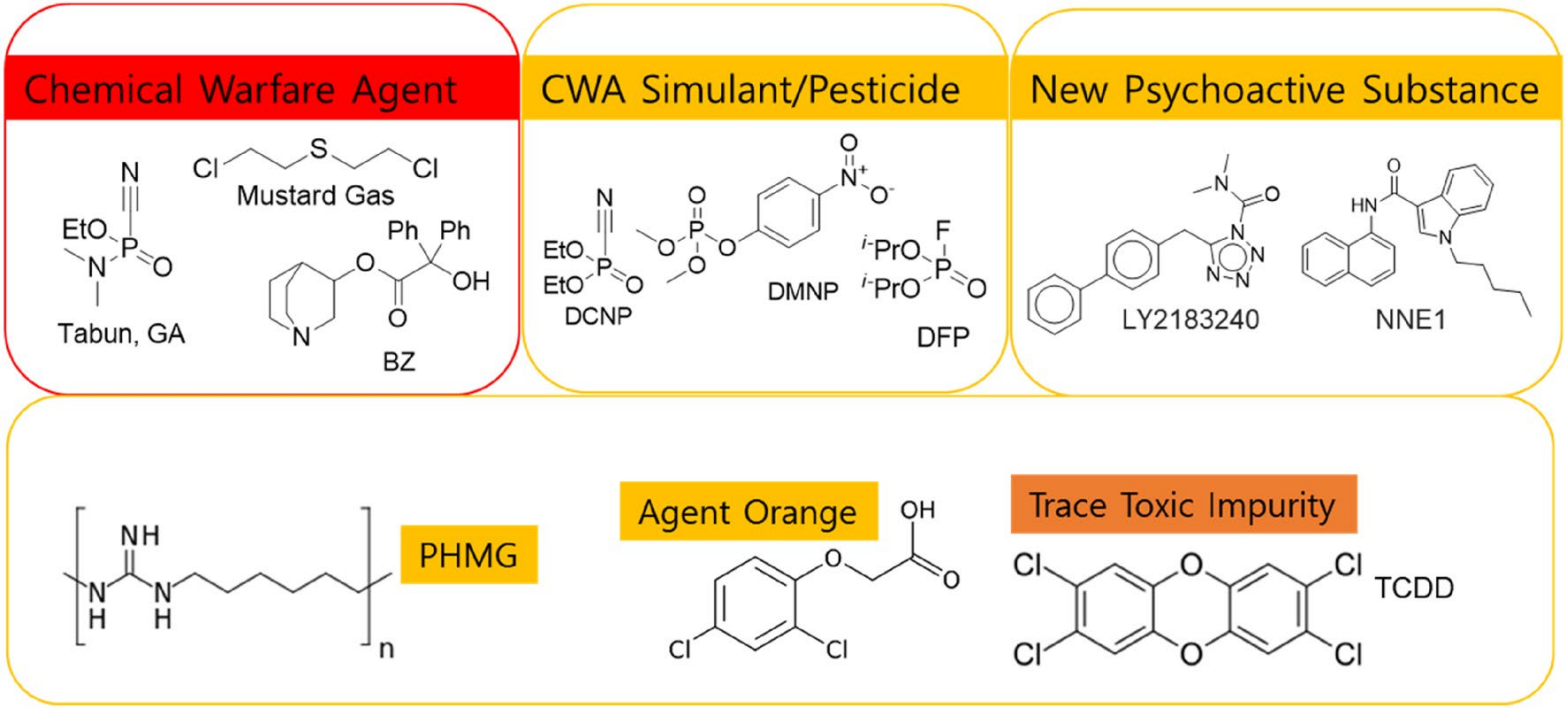
Chemical threats include chemical warfare agents (CWAs) having assigned NATO codes, CWA stimulants, new psychoactive substances (NPSs) and chemical hazards such as PHMG (sterilizer) and TCDD.

Chemical warfare agents (CWAs) and hazardous chemicals threaten chemical safety^1,2^. Prior to the chemical weapons convention, CWAs were intentionally invented and synthesized for military operations. Nowadays, there are concerns about unintentional CWA inventions along with their unexpected accidents through (1) synthetic chemistry related to known CWAs (eg. organophosphorus derivatives)^2,3^ or (2) chemistries for therapeutic drugs (eg. BZ assigned code by NATO) and illegal drugs^4^. Serial terrors such as Sarin in Japan in 1994, VX in Malaysia in 2017, and Novichok (non-declared agent) in Syria in 2018, make the concerns about chemical weapons feasible fears^5^. Moreover, some harmful chemicals (as shown in Fig. 1) were not registered in the CWA list of organizations for the prohibition of chemical weapons (OPCW) but have resulted in devasting causalities, and the tragedies are still going on: (1) ethoxyethyl guanidinium (PGH)/Polyhexamethylene guanidine (PHMG), ingredients of Reckitt Benckiser sterilizers, which resulted in disinfectant deaths of babies and pregnant women in South Korea^6,7^, and (2) TCDD, a trace impurity of Agent Orange (herbicide and defoliant chemical) during the Vietnam War, which has promoted epigenetic transgenerational inheritance of diseases^8,9^.

**Figure 2:**
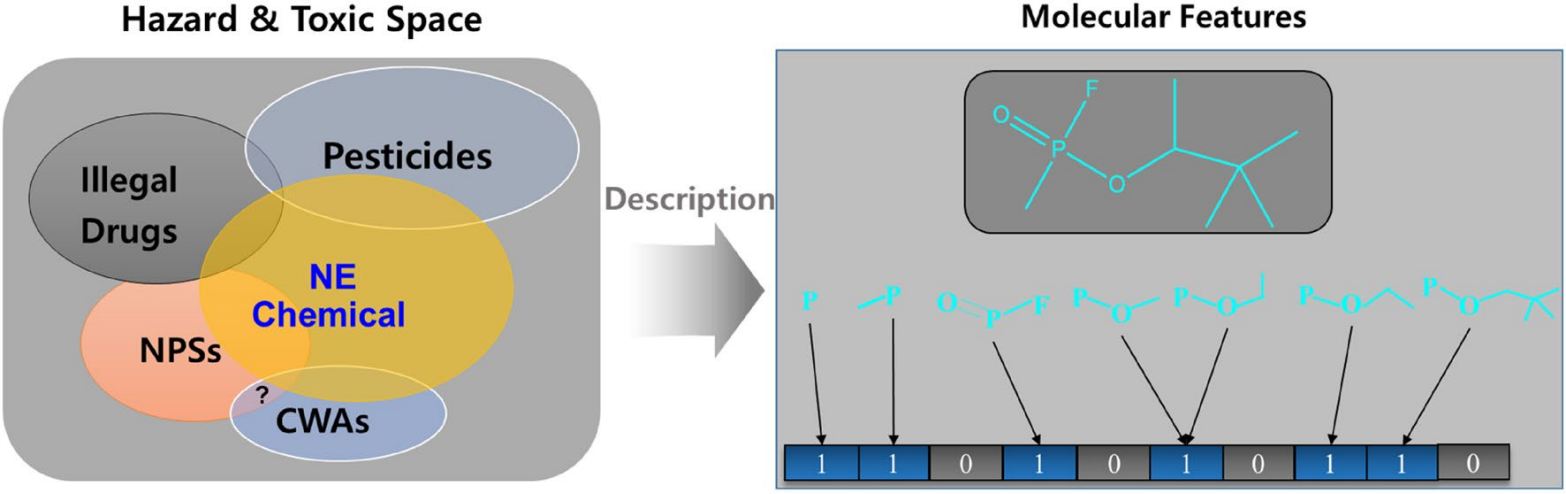
Conceptual presentation of hazard and toxic space and molecular featurization of chemicals in the space.

For chemical safety, humans have built regulations or systems to control the risk resulting from harmful chemicals^10–12^. With such systems, the detection of hazardous agents or their detoxification technologies have been continuously developed^13–16^. Despite the history, the upcoming rate of harmful agents is more rapid than the rate to make a regulation or a detection technology. For example, more than 450 new psychoactive substances (NPSs) or designer drugs, which were designed to mimic the pharmacological effects of known illegal drugs could avoid a regulation of illegal drugs and/or detection in standard drug tests, have been monitored from 2014 to 2017^17–19^. During these periods, any system for safety could not suitably and timely control the NPSs: their identification and detection, evaluation of their toxicity, and establishment of a regulation^20^. Naturally, chemical hazards or toxic substances undefined in a system cannot be prevented, recognized, or controlled^21^. Thus, harmful and hazardous ‘not existing yet but upcoming chemicals (NE chemicals)’ should be pre-defined in advance for the risk assessment. However, the prediction of ‘not-existing’ is vague and indefinite. Fortunately, when a machine learns the structures and properties of known harmful chemicals and analyzes their relationships, the learned relationship can theoretically suggest a pattern of NE chemicals^22^. In other words, a part of the hazard and toxic space can be defined by using molecular features (variables) of known chemicals (Fig. 2). As ‘chemical space’ means which encompasses all possible small molecules^23^, a hazard and toxic space means which encompasses all possible hazardous and toxic chemicals and was named. More desirably, if the definition is ideally achieved, it can be used for preventive regulation. With this consideration, we have tried to define a part of the hazard and toxic space using cholinergic meta-predictors. In this study, the space of pan-cholinergic agents is a priori defined by their molecular structures, and then the cholinergic pattern of nerve agents as CWAs in the space is learned by a convolutional neural network (CNN). The former is the generation of cholinergic meta-predictors and the latter is the CWA detection based on the meta-predictors.

## Results and discussion

### Design of meta-predictor for cholinergic pattern

**Figure 3:**
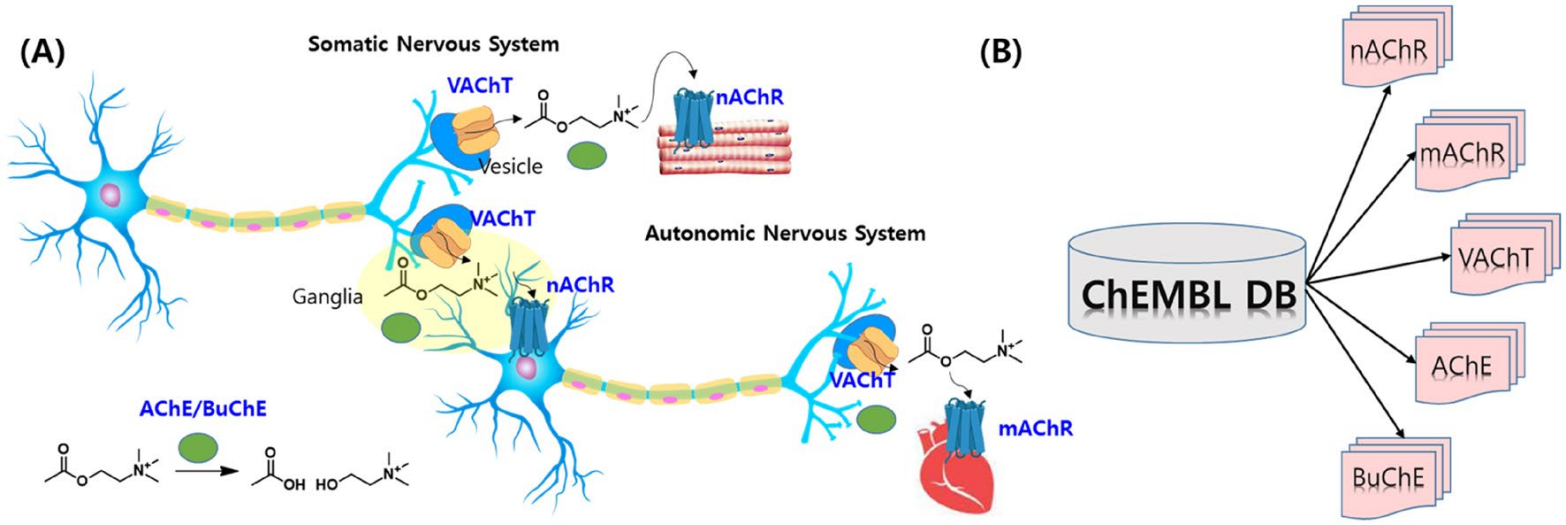
Description of cholinergic space in this study. (**A**) The location and roles of cholinergic targets in the nervous system. (**B**) Data collection of cholinergic agents from the ChEMBL database. All agents were extracted using respective cholinergic targets as MySQL queries. *nAChR* nicotinic acetylcholinesterase receptor, *mAChR* muscarinic acetylcholinesterase receptor, *VAChT* vesicular acetylcholine transporter, *AChE* acetylcholinesterase, *BuChE* butyrylcholinesterase.

For a predictive model, predictor variables and dependent variables are generally chosen (or selected after manipulation) from variables of raw data. However, there was no common information between CWAs and known cholinergic agents, and a toxicity index was rarely available^1,2,16,24^. The available data on cholinergic agents were their structures and cholinergic activities (Fig. 3 and Table S1). Meanwhile, the only common known information about CWAs and harmful agents was molecular structure. Expectedly, linking between CWAs and cholinergic data didn’t produce any common variable. Thus, a practical problem was how to create a unified descriptor (predictor variable) of the chemicals from the limited data. To define a unified descriptor, an important property of hazard and toxic agents is their toxicity profile, together with molecular mechanics, to lead to rescue from toxicity. Notably, the in-depth mechanism of respective toxicity is not clear for most agents and is different from each other. In CWAs, while some nerve agents show high structural congenericity, the structure of 3-quinuclidinyl benzilate (NATO code: BZ) is very dissimilar to those of other CWAs and an outlier in chemical structures of CWAs. Fortunately, nerve agents present relatively more consistent mechanisms based on acetylcholinesterase (AChE) rather than other CWAs such as blister agents, asphyxiants, choking (pulmonary damaging) agents, incapacitating agents, lachrymating agents, and vomit agents^1,2,25,26^. It is well-known that nerve agents and organophosphorus inhibit AChE at cholinergic synapses, thereby inhibiting the degradation of acetylcholine (Fig. 3A). Accumulation of the released acetylcholine causes end-organ overstimulation, which is recognized as a cholinergic crisis^1^.

**Figure 4:**
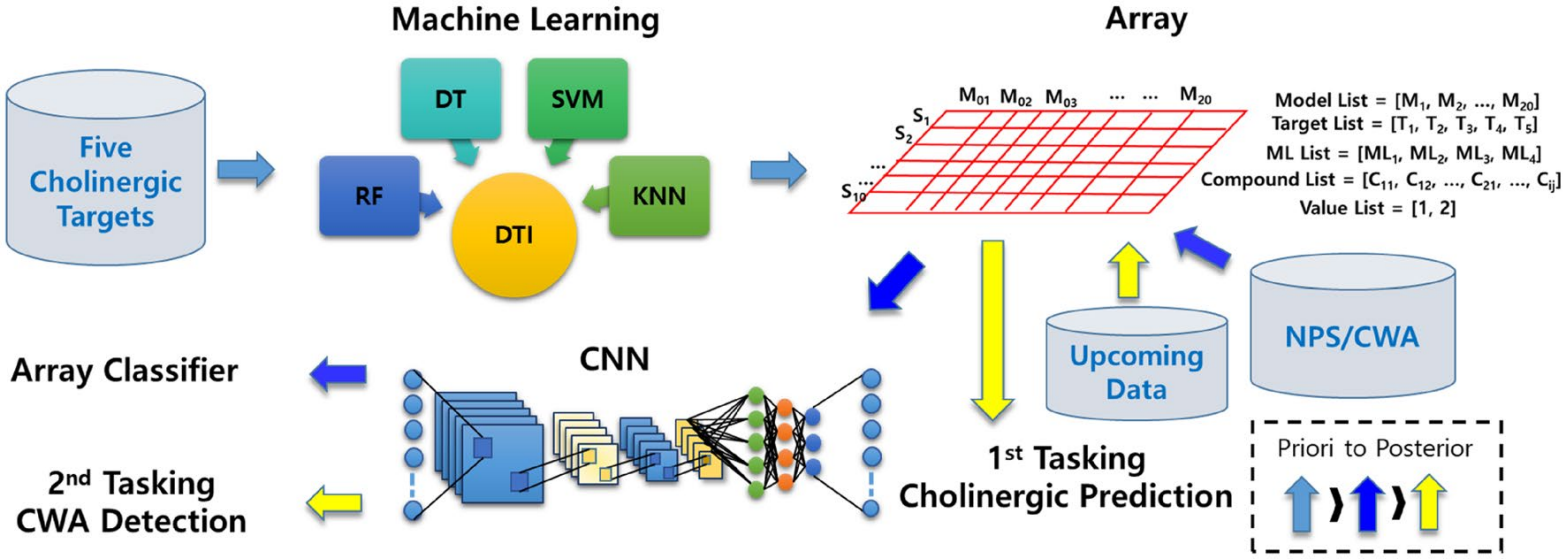
Design of meta-predictor for multi-tasking and data workflow. Pale sky-blue arrow: data flow for building cholinergic DTI models, blue arrow: data flow for building array classifier, yellow arrow: the flow of upcoming data for multi-task. Model list = [M1, M2, …, M20], target list = [T1, T2, T3, T4, T5], ML list = [ML1, ML2, ML3, ML4], seed list = [S1, S2, …, S10], compound list = [C11, C12, …, C21, …, Cij], and value list of predictors =  [1, 2].

Thus, the limited knowledge motivated us to investigate hazard and toxic spaces in terms of their cholinergic effects on the nervous system (of Fig. 3). Notably, the aim of this study was not only cholinergic DTI prediction of individual chemicals but also the detection of CWA from NE chemicals using cholinergic patterns of known chemicals. For this purpose, we designed a meta-predictor to describe the patterns using the structure–activity relationship (SAR) of cholinergic agents (Fig. 4). To our best knowledge, before this study, meta-predictor or meta-learning studies of bio-/chemo-informatics data (1) use homogenous methods iteratively^27^, (2) explicitly adjust weights of element predictors^28,29^, or (3) linearly combine element predictors^29^. More notably, while such known studies used the same dataset for training both predictors and meta-predictors, this study used two heterogenous datasets (cholinergic data in ChEMBL for predictors, and CWA/NPS out of ChEMBL for meta-predictors). We designed our meta-predictor as shown in the below equations. While a predictor, $f$ (of Eq. 1) used data and parameters as input, a meta-predictor, $g$ also used element predictors, $f_{ij}^{{T^{\prime}}}$ in $\left[ {i \times j} \right]$ shaped array. Thus, authors called them ‘meta-predictors’.

1 $$f = L \left( {T, \mathop{w}\limits^{\rightharpoonup} } \right)$$

2 $$g = CNN \left( {f_{ij}^{T\prime } , \overset{\lower0.5em\hbox{$\smash{\scriptscriptstyle\rightharpoonup}$}}{{w_{ij}^{\prime } }} } \right)$$

$$f:predictor, g:meta - predictor, L:machine\; learning\; method$$

$$T:training\;set(ChEMBL),T^{\prime } :training\;set(CWA/NPS), \mathop{w}\limits^{\rightharpoonup}, {\mathop{w}\limits^{\rightharpoonup}}^{\prime}:{\text{vector}}\;{\text{of}}\;{\text{parameters}}$$

Practically, first of all, the biochemical activities of cholinergic agents were embedded together with the molecular descriptors for a machine to learn the SAR. Secondly, the experimental activity data of ChEMBL (a public database) disciplined the machines to judge the relationship between the five cholinergic targets and chemicals, which is called drug target interaction (DTI). The trained DTI models of Fig. 4 (200 classifiers of four type machines, ten differently divided data, and five targets) were internally and externally validated to elucidate the binominal cholinergic patterns (active/inactive) of a chemical. Thirdly, the cholinergic patterns of known CWAs and NPSs as harmful agents were predicted by the 200 binary classifiers, and the predicted values were transformed into an array type data as shown in Fig. 4. Finally, the predicted array data was used as meta-predictors to build the CWA detection model. Even if real cholinergic patterns of these harmful chemicals are unknown, a chemo-centric approach allowed us to infer the pattern. The chemo-centric approach means if two similar molecules are likely to possess similar properties, they can share biological targets or may show similar pharmacological profiles^30–35^. Notably, this study used only two types of real data: chemical structures of all chemicals (ChEMBL, CWAs, and NPSs) and cholinergic activities of ChEMBL chemicals (Fig. 3B).

### Robust DTI classification models for meta prediction

**Figure 5:**
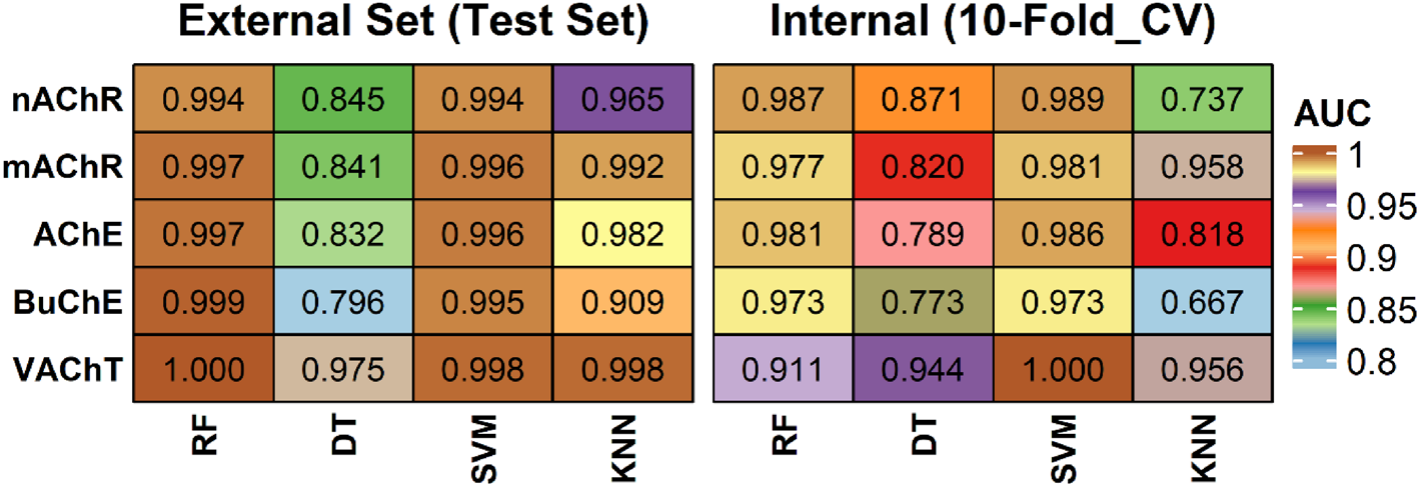
Statistical performance of DTI classification models. The area under receiver operating characteristic curve (AUC) was calculated in external and internal validation of respective targets.

To realize the designed meta-predictor, two types of 2D molecular fingerprints (FCFP, ECFP) captured the molecular structures of all cholinergic agents^36^. These extended-connectivity and functional-class fingerprints are well-known molecular representations, which precisely describe molecular structure and functional groups (groups of atoms having their own characteristic properties) in a molecule and show their competent performance in drug design and large-scale prediction^36^. Thus, ECFP and FCFP were used to describe the cholinergic SAR under machine learning (ML) algorithms of random forest (RF), support vector machine (SVM), decision tree (DT), and k-nearest neighbor (KNN)^37–39^. The DTI model was trained for each cholinergic target of acetylcholinesterase (AChE), butyrylcholinesterase (BuChE), nicotinic acetylcholinesterase receptor (nAChR), muscarinic acetylcholinesterase receptor (mAChR), and vesicular acetylcholine transporter (VAChT)^40^. Firstly, statistical performance for the nAChR classifier was evaluated (Table 1 and Table S2). Expectedly, the receiver operating characteristic (ROC) plots of nAChR classifiers demonstrated the robust predictability irrespective of data division into training and test (Table S2 and Fig. S2). When Area Under ROC (AUC) of test data was compared, RF, SVM, and KNN models (AUC: 0.961–0.998) produced AUC higher than DT (AUC: 0.739–0.889). Furthermore, we applied other statistical metrics including accuracy, F1 score, and Matthews correlation coefficient (MCC), which informative and truthful scores in evaluating binary classifications than accuracy and F1 score. Notably, the MCC values of every model were reliable (Test: MCC ~ 0.438–0.978, Train: 0.474–0.956), and the MCC values of test sets were at par with those of train sets. Secondly, the learning of the mAChR dataset followed a similar pattern to nAChR models, along with AUC of 0.807–0.998 and MCC of 0.608–0.974 (Table 1 and Table S3). The mAChR models produced slightly higher predictive performance than the nAChR models. The overall DT model presented a lower performance than RF, SVM and KNN models. Thirdly, BuChE models also showed reliable prediction performance with AUC of 0.771–1.000 and MCC of 0.420–0.986 and slightly lower than the classification models of nAChR and mAChR (Table 1 and Table S5). Fourthly, we further analyzed the classification metrics from AChE models. Despite the large data size (n = 3098), the classification performance revealed at par performance for AUC of 0.774–0.999 (Table 1 and Table S4). Finally, VAChT models of the smallest dataset outperform those of nAChR, mAChR, AChE, and BuChE (Table 1 and Table S6). To visualize the predictive power of the cholinergic DTI models, the best performing models were described by ensemble-AUC values (Fig. 5 and Table S7).

**Table 1:** The classification performance of selected best model based on ensemble-AUC for train and test set.

Target	ML	AUC	MCC	ACC	F1-Score
nAChR	RF	0.994 (0.987)	0.918 (0.975)	0.959 (0.987)	0.959 (0.987)
	DT	0.845 (0.871)	0.678 (0.764)	0.836 (0.871)	0.824 (0.854)
	SVM	0.994 (0.989)	0.936 (0.978)	0.968 (0.989)	0.968 (0.989)
	KNN	0.741 (0.737)	0.551 (0.558)	0.741 (0.737)	0.791 (0.792)
mAChR	RF	0.997 (0.977)	0.952 (0.954)	0.976 (0.977)	0.976 (0.977)
	DT	0.841 (0.820)	0.673 (0.642)	0.837 (0.820)	0.834 (0.813)
	SVM	0.996 (0.981)	0.959 (0.962)	0.979 (0.981)	0.979 (0.981)
	KNN	0.992 (0.958)	0.911 (0.917)	0.956 (0.958)	0.955 (0.958)
AChE	RF	0.997 (0.981)	0.942 (0.962)	0.971 (0.981)	0.971 (0.981)
	DT	0.832 (0.789)	0.627 (0.597)	0.808 (0.789)	0.824 (0.813)
	SVM	0.996 (0.986)	0.943 (0.972)	0.971 (0.986)	0.972 (0.986)
	KNN	0.982 (0.818)	0.704 (0.683)	0.832 (0.818)	0.856 (0.846)
BUChE	RF	0.999 (0.973)	0.949 (0.948)	0.974 (0.973)	0.974 (0.973)
	DT	0.796 (0.773)	0.523 (0.566)	0.761 (0.773)	0.760 (0.799)
	SVM	0.995 (0.973)	0.961 (0.947)	0.980 (0.973)	0.980 (0.973)
	KNN	0.909 (0.667)	0.408 (0.447)	0.643 (0.667)	0.737 (0.750)
VAChT	RF	1.000 (0.911)	0.702 (0.915)	0.830 (0.956)	0.887 (0.957)
	DT	0.975 (0.944)	0.953 (0.934)	0.976 (0.967)	0.976 (0.966)
	SVM	0.998 (1.000)	0.991 (1.000)	0.995 (1.000)	0.991 (1.000)
	KNN	0.998 (0.956)	0.953 (0.934)	0.976 (0.967)	0.977 (0.967)

*ACC* Accuracy, *MCC* Matthew’s Correlation Coefficient, *RF* Random Forest, *DT* Decision Tree, *SVM* Support Vector Machine, *KNN* K-Nearest Neighbor, *nAChR* Nicotinic Acetylcholinesterase Receptor, *mAChR* Muscarinic Acetylcholinesterase Receptor, *AChE* Acetylcholinesterase Enzyme, *BuChE* Butyrylcholinesterase Enzyme, *VAChT* Vesicular Acetylcholine Transporter. The values in parenthesis belong to the test set. The best model was selected based on the ensemble-AUC (Table S7).

### Multi-task of array classifiers and performance

**Figure 6:**
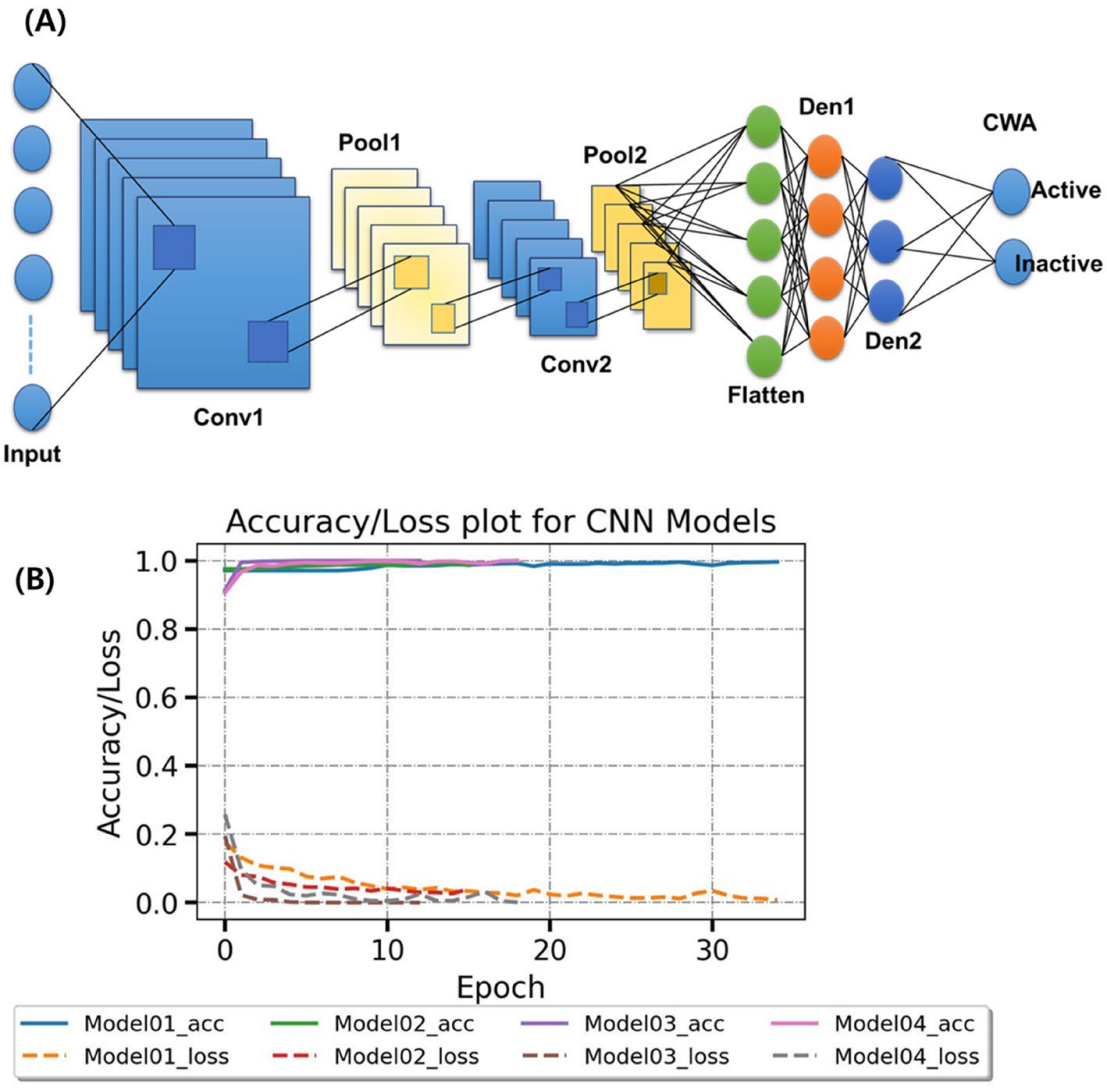
Training of the three different shaped array classifiers (2D, 3D, and reshaped 3D). (**A**) CNN architecture in this study. (**B**) Robust training of the CNN models with early stopping via callback. X-axis: the number of epochs (training unit), y-axis: accuracy or loss values (the gap between real and prediction), which were calculated by a loss function according to data sampling (Model 01: native, Model 02: removal of duplicated array values from Model 01 data, Model 03: SMOTE oversampling of Model 01 data, Model 04: SMOTE oversampling of Model 02 data).

**Figure 7:**
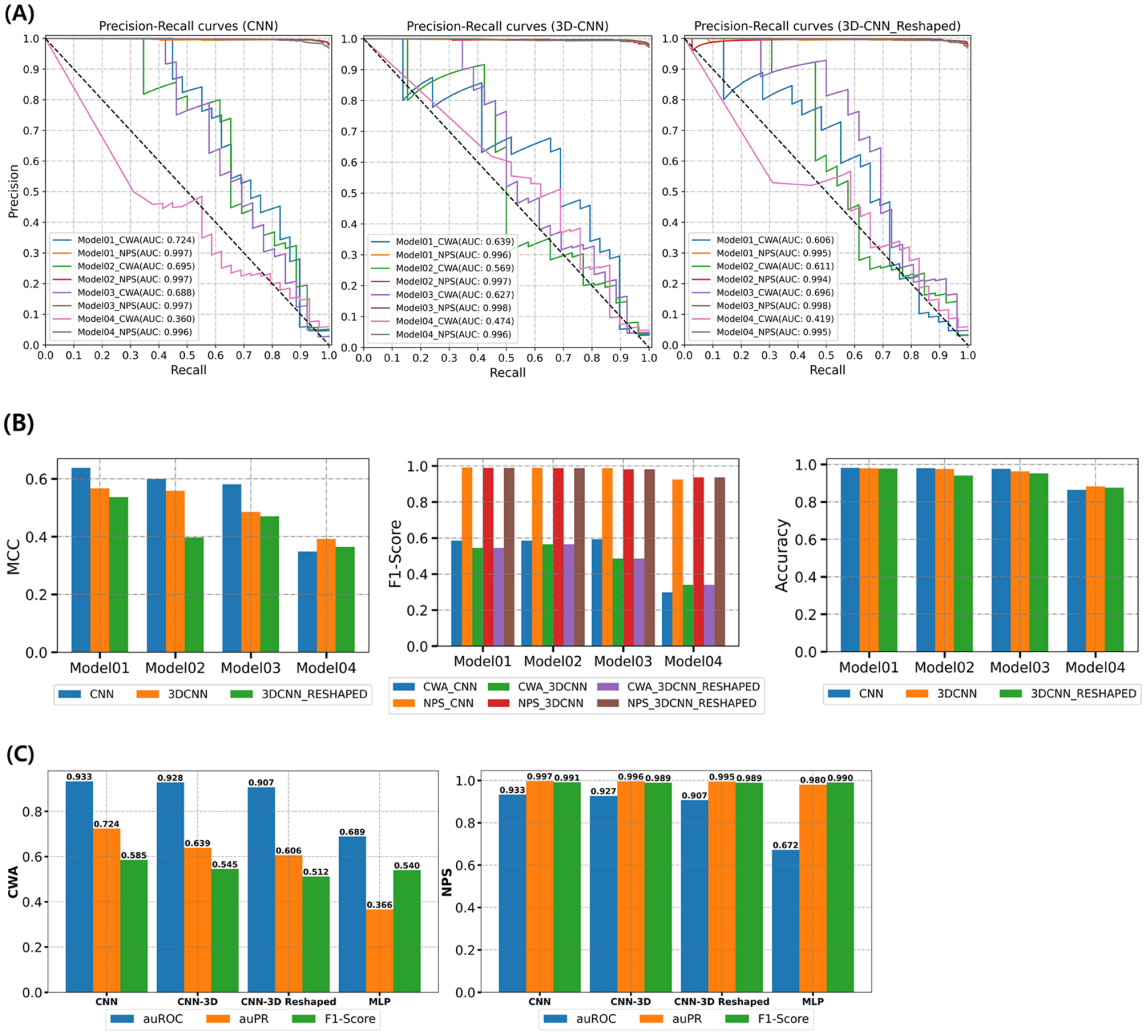
CWD detection performance of the three different shaped array classifiers according to data sampling (Model 01 to 04). (**A**) Precision-Recall curve of CNN models, (**B**) Performance of CNN models (MCC, F1-Score, and Accuracy), and (**C**) comparison with multiple layer perceptron as a baseline model.

The first task of the built array model is predicting cholinergic activities of ‘out-of-set (neither training nor test set)’ molecules on nAChR, mAChR, VAChT, AChE, and BUChE (Fig. 4). For the purpose, every cholinergic DTI classifier was already validated in the prior section. Clearly, CWAs and none CWAs consisting of NPSs and designer drugs^19^ are out of ChEMBL cholinergic data^40^, neither training nor test data. Cholinergic patterns of the CWAs and none CWAs were predicted to play the role of meta-predictors for the second task. The second task of the array model is judging the chemical warfare likeness of ‘out-of-set’ molecules. For this purpose, the discrimination between CWAs and none CWAs was learned by a CNN algorithm. CNN is a popularly used deep learning framework for object recognition tasks, object tracking, pose estimation, text detection and recognition, visual saliency detection, action recognition, scene labeling^41^. LeNet of LeCun et al.^42^ and AlexNet of Hinton et al.^43^ initialized the popularity of CNN in the field of computer vision. GoogleNet^44^, VGGNEt^45^, ResNet^46^, and so on elaborated CNN architecture (e.g., batch normalization, filter, residual function) improved prediction accuracy. Despite the difference in data size, our meta-predictors have the same property as a binary pixel array with MNIST hand-written data (28 × 28 pixels with two colors), which is a representative dataset of CNN models^47^. The common property made us benchmark the image-based learning of MNIST data. Firstly, our meta-predictors were converted to the 2D array of a 5 × 4 shape for CNN learning. After the investigation, the architecture of Fig. 6A (see also Fig. S9) was chosen for the best learner. As our expectation, the 2D array reliably detected CWAs from large NPS data. During the learning along with the increased epoch, accuracy and loss values reached their optimal values and retained the values (Fig. 6B). With the encouraging results, we tried to adjust the data imbalance between CWAs and non-CWAs through over-sampling and under-sampling (the removal of data showing duplicated array values). As shown in Fig. 7, when imbalanced native data (Model 01) was compared with balanced over-sampled data (Model 03), statistical metrics showed the deviation with a slight decrease, but the area under the precision-recall curve (AUPR) values of Fig. 7A were still comparable between native (imbalanced) and over-sampled data (balanced) to prove that these statistical values did not simply result from data imbalances. The Matthews correlation coefficient (MCC), F1-score, and accuracy (Fig. 7B) also supported that the SMOTE (over-sampling) confirmed the ability to find CWAs^48^. Furthermore, the two types of sampling allowed us to evaluate 2D or 3D array classifiers of different shapes. When we re-shaped the 2D array from [50 × 4] to [40 × 5], the detection ability steeply decreased to reveal the importance of how to arrange element predictors. If some data shows a dependency on the order between its variables (element predictors), the data can be called sequential. Meanwhile, when we converted the 2D array into 3D arrays, surprisingly, image-based learning of [10 × 5 × 4] shape improved AUPR, MCC, and F1-score of the worst ‘Model 04’ and decreased the performance gap between different data (Fig. 7). When the 3D array was reshaped into [5 × 10 × 4], the improvement of these statistical values was also retained. Moreover, multi-layer perceptron (MLP) model was built from the training data of the CNN model with the same number of layers. The MLP model as a baseline showed very inferior precision and a lower F1 score than the best CNN model. In detail, while two CNN models were superior to the MLP model, still shape of the array was still important to give how much better performance than MLP (Fig. 7C).

Based on the statistical validation of Fig. 7 and Table S8, the array classifiers are ready for CWA detection of NE chemicals. Obviously, this predictive model for chemical threats under the chemo-centric assumption is arguable due to the available data and impossible experimental validation. However, such a trial is not the only one. For example, the OECD also developed the QSAR model toolbox and has provided it for risk assessment^10^. Although typical QSAR models can guarantee high precision within their prediction domain, the QSAR models have the limitation of a domain beyond the congeneric series set. For example, when the six acute toxicity models of the OECD QSAR Toolbox predicted CWAs, considerable CWAs could not return the predicted values due to out of prediction domain and gave a failure rate of ca. 50% (Table 2). Thus, innovative studies should overcome the limitation of a domain. Moreover, QSAR models generally cannot be built using an ab initio method. Unfortunately, toxicity index of CWA data is rare, not enough to build a reliable predictive model. Obviously, although a classification model can be built, the prediction domain is still biased to organophosphorus. Thus, we suggested cholinergic meta-predictors in order to investigate the make-up of the current data limitation. The notable difference between this study and typical QSAR models is the definition of the dependent variable and independent variable. Typical QSAR models use ‘experimental activity/toxicity index’ as a dependent variable and ‘chemical structure and their descriptors’ as independent variables. Meanwhile, the CNN model of this study did not use either chemical structures or their molecular descriptors. The encoding of chemical structures was replaced with cholinergic meta-predictors. Because current information on the mechanism of CWAs is enriched in AchE and cholinergic effects, this study only described cholinergic patterns to detect chemical threats. In the future, if data is updated, this methodology could be applicable to other pharmacological effects of known harmful chemicals such as brain monoacylglycerol (MAG) lipase activity and endocannabinoid degrading enzyme, fatty acid amide hydrolase (FAAH), which are recently reported toxicity mechanisms of organophosphorus pesticides^2,16^. Even if the MAG and FAAH inhibition of the insecticides were reported, such a trial would be more feasible after updating the data (of MAG or FAAH agents) as much as those of cholinergic agents.

**Table 2:** Acute toxicity QSAR models for human health hazards and toxicity prediction of CWAs.

OECD-QSAR model	Failure rate^a^
Acute toxicity in mouse (intraperitoneal)	0.58
Acute toxicity in mouse (intravenous)	0.48
Acute toxicity in mouse (oral)	0.59
Acute toxicity in mouse (subcutaneous)	0.53
Acute toxicity in rat (intraperitoneal)	0.56
Acute toxicity in rat (oral)	0.36

CWA data of CNN models was used for the prediction. Every CWA should be inserted through query search in IUCLID databased implemented in the OECD QSAR Toolbox.

^a^The failure rate was % ratio of the counted ‘no value’ in each predictive model. No value was produced with ‘out-of-domain’, ‘not applicable’, and none mentioned reason.

## Conclusion

Despite extremely imbalanced data, the cholinergic pattern of CWAs was learned through array-type meta-predictors to achieve acceptable predictive performance. Furthermore, the learning allows multi-tasking for a chemical: DTI prediction for five cholinergic targets under four ML algorithms and CWA detection under the CNN algorithm. While the former task was verified through the internal and external validation of the respective DTI classifier, the latter task was validated using CWA and non-CWA. Notably, this study suggests a new method to describe harmful agents having limited information for their quantitative structure–toxicity relationship. Thus, it contributes to the research controlling and predicting chemical threats from NE chemicals in the recent future.

## Methods

### Dataset collection and manipulation

Any machine learning algorithm inextricably relies on the structure and reported activity data. In recent years, the ChEMBL databases have become a primary source for retrieving chemical data for machine learning applications. Herein, the ChEMBL database version 24^49^ was selected to retrieve the structural and property data of cholinergic agents (nAChR, mAChR, VAChT, AChE, and BUChE) with the MySQL query consisting of molecular structures (canonical smiles), activity ID, standard values of inhibitory activities with standard relation and standard unit (nanomolar), assay ID, and target ID. In addition, the molecular structures of CWAs and NPSs were collected from literature^1,2,19^ and NPS-datahub^50^. Every manipulation of data (sorting, merging, cleaning of duplicated data, and binominalization) was conducted by the KNIME Analytic Platform^51^. The supplementary section describes the composition of chemicals in each target. In brief, a total number of 1818, 6944, 3098, 1382, 302, 95, and 3126 chemicals belonging to nAChR, mAChR, AChE, BuChE, VAChT, CWA, and NPSs were selected respectively.

#### MySQL query in ChEMBL DB

Select x.molregno,canonical_smiles, activity_id,y.assay_id, standard_value, standard_relation, standard_units, i.tid, k.target_type, k.pref_name, k.organism From compound_structures x, activities y, assays i, target_dictionary k.

Where x.molregno = y.molregno and y.assay_id = i.assay_id and i.tid = k.tid and k.tid = 10532 INTO outfile "chembl_target_BuChE.csv" fields terminated by ',' lines terminated by '/n';

### Molecular descriptor generation

Eight 2D molecular fingerprints of every chemical data were generated with (1) two types, extended-connectivity fingerprint (ECFP) and functional-class fingerprint (FCFP), and (2) 4 different diameters (0, 2, 4, 6) under a fixed 1024-bit vector size. Notably, ECFP captures precise atom properties (e.g. atomic number, charge, hydrogen count, etc.), whereas FCFP captures functional (pharmacophoric) features (i.e. hydrogen donor/acceptor, polarity, aromaticity, etc.) of the atoms in a molecule. The CDK toolkit^52^ was used for both fingerprint calculations. The generated fingerprints were split and combined with respective binominal activity values into an embedded data matrix for learning.

### Building classification models and validation

Four machine learning algorithms (random forest, decision tree, support vector machine, and k-nearest neighbor) applied on the data matrix with 10 different random seed numbers to build a classification model in the classification and regression training (CARET) package of the R environment. Every model was internally and externally validated in the condition of a 70:30 division ratio between training and test and k-fold (k = 10) cross-validation methods. In brief, in k-fold cross validation, the input data is randomly partitioned into k-equal size subsamples. One of the k subsamples is kept as validation data for testing the model, while the remaining k-1 subsamples are used as training data. This k-fold cross-validation procedure is then repeated k times (the folds), with each of the k subsamples used exactly once as the validation data.

### Array classifier-CNN architecture

The built models generated meta-predictors (meta-data) of 200 binary bits (5 cholinergic targets × 4 machine learning methods × 10 seed numbers). The metadata was embedded through several shape arrays of ([50 × 4], [5 × 10 × 4], [10 × 5 × 4]). The CNN model, which is composed of different layers of convolutional, pooling, flatten, and dense layers was built with the hyperparameters of maximum of 100 epochs, a batch size of 32 and a learning rate of 0.01 with the Adam optimizer^53^. The EarlyStopping criteria were introduced to prevent the CNN models from being over-fitting and to terminate the learning early. The ‘Softmax’ activation function was used to define the probability distribution of the chemical warfare likeness^54^. The learning performance and robustness were measured by accuracy and loss values as the epoch number increased. Binary cross-entropy was used as the loss function to measure the deviation between the predicted and actual class values.

$${\text{Loss}} = { } - \frac{1}{m + n}\left[ {\mathop \sum \limits_{i}^{m} \log \left( {f\left( {x_{i}^{ + } } \right)} \right) + \mathop \sum \limits_{i}^{n} \log \left( {1 - f\left( {x_{i}^{ - } } \right)} \right)} \right]$$

### Evaluation of predictive model

The performance of each models was evaluated using three classification metrics i.e. Matthews correlation coefficient (MCC), accuracy, the area under the receiver operating characteristic curve (AUC) based on true positive (TP), true negative (TN), false positive (FP), false negative (FN). These metrics evaluate the statistical performance and robustness of built models.

$${\text{Accuracy }}\left( {{\text{ACC}}} \right){ } = \frac{{\left( {{\text{TP}} + {\text{TN}}} \right)}}{{\left( {{\text{TP}} + {\text{FP}} + {\text{TN}} + {\text{FN}}} \right)}}$$

$${\text{Precision}} = { }\frac{{{\text{TP}}}}{{{\text{TP}} + {\text{FP}}}}$$

$${\text{F}}1\;{\text{ Score}} = \frac{{2{ } \times { }\left( {{\text{TP }}/({\text{TP}} + {\text{FN}})} \right){ } \times { }\left( {{\text{TP }}/({\text{TP}} + {\text{FP}})} \right)}}{{\left( {{\text{TP }}/({\text{TP}} + {\text{FN}})} \right) + { }\left( {{\text{TP }}/({\text{TP}} + {\text{FP}})} \right)}}$$

$${\text{MCC}} = { }\frac{{{\text{TP}} \times {\text{TN}} - {\text{FP}} \times {\text{FN }}}}{{\sqrt {\left( {{\text{TP}} + {\text{FP}}} \right){ }\left( {{\text{TP}} + {\text{FN}}} \right){ }\left( {{\text{TN}} + {\text{FP}}} \right){ }\left( {{\text{TN}} + {\text{FN}}} \right)} }}$$

$${\text{AUC}} = { }\frac{{{\text{TPR}} + {\text{TNR}}}}{2}$$

$${\text{Recall}} = {\text{TPR }}\left( {{\text{True}}\;{\text{ Positive}}\;{\text{ rate}}} \right) = { }\frac{{{\text{TP}}}}{{{\text{TP}} + {\text{FN}}}}$$

$${\text{TNR }}\left( {{\text{True}}\;{\text{ Negative}}\;{\text{ Rate}}} \right) = { }\frac{{{\text{TN}}}}{{{\text{TN}} + {\text{FP}}}}$$

### Ethics approval and consent to participate

Every author accepted ethical standards of a genuine research study.

## Supplementary Information

Supplementary Information 1.

Supplementary Information 2.

## Data Availability

Python code, and refined data will be available in GitHub. https://github.com/college-of-pharmacy-gachon-university/Array_Classifier.
