# Supplementary material for: Prediction of chemical warfare agents based on cholinergic array type meta-predictors
Source: Sci Rep. 2022 Oct 6;12:16709. doi: 10.1038/s41598-022-21150-2 (PMC9537167; doi:10.1038/s41598-022-21150-2)
Supplement: Supplementary file 1 — Supplementary Information 1. [file 41598_2022_21150_MOESM1_ESM.zip › Array_Classifier-main/script/MLP_Baseline_Model_Without_SMOTE.html]

Model\_01\_MLP\_Baseline\_Model\_Without\_SMOTE\_Trial


In [1]:

```
import warnings
warnings.filterwarnings("ignore")
warnings.simplefilter(action='ignore', category=FutureWarning)

import os
import numpy as np
import random as python_random
import tensorflow as tf 
SEED=56789
np.random.seed(SEED)
python_random.seed(SEED)
tf.compat.v1.random.set_random_seed(SEED)
os.environ['PYTHONHASHSEED']=str(SEED)
import pandas as pd
from PIL import Image
import sys
import tensorflow.keras
from tensorflow.keras import backend 
from tensorflow.keras.models import Sequential, load_model 
from tensorflow.keras.layers import LSTM, Dense, RepeatVector, Masking, Dropout, Flatten, Conv2D, MaxPooling2D
from tensorflow. keras.utils import plot_model 
from sklearn import metrics
from sklearn.metrics import classification_report, confusion_matrix, accuracy_score, f1_score, precision_score, recall_score
from sklearn.metrics import roc_curve, auc, plot_precision_recall_curve, average_precision_score, precision_recall_curve
from sklearn.model_selection import train_test_split
from tensorflow.keras.callbacks import EarlyStopping
import matplotlib.pyplot as plt 
from sklearn.neural_network import MLPClassifier
from sklearn.metrics import roc_auc_score, accuracy_score, classification_report, make_scorer
from sklearn.metrics import plot_roc_curve, confusion_matrix, auc, plot_precision_recall_curve
```

In [2]:

```
classdata = pd.read_csv('200_Model_Comibined_Data_without_Smote.csv')
fps = classdata['FP']
X = []
Y = classdata['CLASS'].values
for i in range(0,len(fps)):
    cmap = {'1': 1, '2': 2}
    data = [cmap[letter] for letter in fps[i]]
    X.append(np.array(data))
    
X_train, X_test, Y_train, Y_test = train_test_split(X, Y, test_size=0.3, random_state=10, stratify=Y) 
Y_train = tf.keras.utils.to_categorical(np.array(Y_train), num_classes=2)
Y_test = tf.keras.utils.to_categorical(np.array(Y_test), num_classes=2)
```

In [3]:

```
estimator = MLPClassifier(hidden_layer_sizes=(7, ), random_state=0)
model_TRTS = estimator
model_TRTS.fit(X_train, Y_train)
    
y_pred_train = model_TRTS.predict(X_train)
y_pred_test = model_TRTS.predict(X_test)
```

In [4]:

```
# Train statistics
acc_tr = accuracy_score(Y_train, y_pred_train)
mcc_tr = metrics.matthews_corrcoef(Y_train.argmax(axis=1), y_pred_train.argmax(axis=1))
f1_score_tr = metrics.f1_score(Y_train, y_pred_train, average='macro')
roc_auc_tr = metrics.roc_auc_score(Y_train, y_pred_train, average='macro')
auPR_tr = average_precision_score(Y_train, y_pred_train, average='macro')    
cm_tr = confusion_matrix(Y_train.argmax(axis=1), y_pred_train.argmax(axis=1))

print();print('MLP Classifier Statistics for Train: ')
print("Accuracy_Train: %.4f" % acc_tr)
print("MCC_Train: %.4f" % mcc_tr)
print("f1_score_Train: %.4f" % f1_score_tr)
print("ROC_AUC_Train: %.4f" % roc_auc_tr)
print("auPR_Train: %.4f" % auPR_tr)
print("Confusion_Matrix_Train:", cm_tr)
print(classification_report(Y_train, y_pred_train))


# Test statistics
acc_ts = accuracy_score(Y_test, y_pred_test)
mcc_ts = metrics.matthews_corrcoef(Y_test.argmax(axis=1), y_pred_test.argmax(axis=1))
f1_score_ts = metrics.f1_score(Y_test, y_pred_test, average='macro')
roc_auc_ts = metrics.roc_auc_score(Y_test, y_pred_test, average='macro')
auPR_ts = average_precision_score(Y_test, y_pred_test, average='macro')    
cm_ts = confusion_matrix(Y_test.argmax(axis=1), y_pred_test.argmax(axis=1))

print();print('MLP Classifier Statistics for Test: ')
print("Accuracy_Test: %.4f" % acc_ts)
print("MCC_Test: %.4f" % mcc_ts)
print("f1_score_Test: %.4f" % f1_score_ts)
print("ROC_AUC_Test: %.4f" % roc_auc_ts)
print("auPR_Test: %.4f" % auPR_ts)
print("Confusion_Matrix_Test:", cm_ts)
print(classification_report(Y_test, y_pred_test))
```

```
MLP Classifier Statistics for Train: 
Accuracy_Train: 0.9823
MCC_Train: 0.6465
f1_score_Train: 0.8017
ROC_AUC_Train: 0.7194
auPR_Train: 0.7125
Confusion_Matrix_Train: [[  30   36]
 [   2 2186]]
              precision    recall  f1-score   support

           0       0.94      0.45      0.61        66
           1       0.98      1.00      0.99      2188

   micro avg       0.98      0.98      0.98      2254
   macro avg       0.96      0.73      0.80      2254
weighted avg       0.98      0.98      0.98      2254
 samples avg       0.98      0.98      0.98      2254


MLP Classifier Statistics for Test: 
Accuracy_Test: 0.9793
MCC_Test: 0.5827
f1_score_Test: 0.7630
ROC_AUC_Test: 0.6805
auPR_Test: 0.6732
Confusion_Matrix_Test: [[ 11  18]
 [  1 937]]
              precision    recall  f1-score   support

           0       0.92      0.38      0.54        29
           1       0.98      1.00      0.99       938

   micro avg       0.98      0.98      0.98       967
   macro avg       0.95      0.69      0.76       967
weighted avg       0.98      0.98      0.98       967
 samples avg       0.98      0.98      0.98       967
```

In [5]:

```
fpr = dict()
tpr = dict()
roc_auc = dict()
for i in range(2):
    fpr[i], tpr[i], _ = roc_curve(Y_test[:, i], y_pred_test[:, i])
    roc_auc[i] = auc(fpr[i], tpr[i])
        
precision = dict()
recall = dict()
average_precision = dict()
    
for i in range(2):
    precision[i], recall[i], _ = precision_recall_curve(Y_test[:, i], y_pred_test[:, i])
    average_precision[i] = average_precision_score(Y_test[:, i], y_pred_test[:, i])
    
precision["micro"], recall["micro"], _ = precision_recall_curve(Y_test.ravel(), y_pred_test.ravel())
average_precision["micro"] = average_precision_score(Y_test, y_pred_test, average="micro")
    
fig, ax = plt.subplots(1,2,figsize=(9,5))
    
for i, classes in zip(range(2), ['Class 0', 'Class 1']):
    ax[0].plot(fpr[i], tpr[i], label=classes+'(AUC: %0.3f)' % roc_auc[i], alpha=1)
ax[0].plot([0, 1], [0, 1],'k--')
ax[0].set_xlim([0.0, 1.01])
ax[0].set_ylim([0.0, 1.01])
ax[0].set_yticks(np.arange(0, 1.1, 0.1))
ax[0].set_xticks(np.arange(0, 1.1, 0.1))
ax[0].set_xlabel('False Positive Rate', fontsize=14)
ax[0].set_ylabel('True Positive Rate', fontsize=14)
ax[0].set_title('ROC curves', fontsize=14)
ax[0].grid(linestyle='-.', linewidth=0.7)
ax[0].tick_params(axis="y", labelsize=12) 
ax[0].tick_params(axis="x", labelsize=12)
ax[0].legend(fontsize=10, loc="lower right")

for i, classes in zip(range(2), ['Class 0', 'Class 1']):
    ax[1].plot(recall[i], precision[i], label=classes+'(AUC: %0.3f)' % average_precision[i], alpha=1)     

ax[1].plot([0, 1], [1, 0],'k--')
ax[1].set_xlim([0.0, 1.01])
ax[1].set_ylim([0.0, 1.01])
ax[1].set_yticks(np.arange(0, 1.1, 0.1))
ax[1].set_xticks(np.arange(0, 1.1, 0.1))
ax[1].set_xlabel('Recall', fontsize=14)
ax[1].set_ylabel('Precision', fontsize=14)
ax[1].set_title('Precision-Recall curves (micro avg :{0:0.3f})'.format(average_precision["micro"]), fontsize=14)
ax[1].grid(linestyle='-.', linewidth=0.7)
ax[1].tick_params(axis="y", labelsize=12) 
ax[1].tick_params(axis="x", labelsize=12)
ax[1].legend(fontsize=10, loc="lower left")
plt.tight_layout()
```

In [ ]:

```

```
