# Supplementary material for: Prediction of chemical warfare agents based on cholinergic array type meta-predictors
Source: Sci Rep. 2022 Oct 6;12:16709. doi: 10.1038/s41598-022-21150-2 (PMC9537167; doi:10.1038/s41598-022-21150-2)
Supplement: Supplementary file 2 — Supplementary Information 2. [file 41598_2022_21150_MOESM2_ESM.docx]

Supporting Information

**Prediction of Chemical Warfare Agents Based on Cholinergic Array Type Meta-Predictors**

Surendra Kumar^a,†^, Chandni Kumari^a,†^, Sangjin Ahn^a,b^, Hyoungrae Kim^c^ and Mi-hyun Kim^a,*^

^a^Gachon Institute of Pharmaceutical Science and Department of Pharmacy, College of Pharmacy, Gachon University, 191 Hambakmoeiro, Yeonsu-gu, Incheon, Republic of Korea. ^b^Department of Financial Engineering, College of Business, Ajou University, Suwon, 16499, Republic of Korea. ^c^Department of Data Management, KEIS, 56 Mullae-ro 20-gil,Yeongdeungpo-gu, Seoul, Republic of Korea

^†^ The authors are co-first authors

^*^Author for correspondence

E-mail: [kmh0515@gachon.ac.kr](mailto:kmh0515@gachon.ac.kr)

**Table of Contents**

| **S. No.** | **Descriptions** | **Page No.** |
| --- | --- | --- |
| **1.** | Table S1: Dataset Description. | S3 |
| **2.** | Figure S1: The workflow to build the SAR classification model. | S4 |
| **3.** | Table S2: The statistical performance of nAChR. | S5 |
| **4.** | Table S3: The statistical performance of mAChR. | S6 |
| **5.** | Table S4: The statistical performance of BuChE. | S7 |
| **6.** | Table S5: The statistical performance of AChE. | S8 |
| **7.** | Table S6: The statistical performance of VAChT. | S9 |
| **8.** | Table S7: The ensemble-AUC based on different machine learning method for different models. | S10 |
| **9.** | Figure S2: The statistical performance (ROC plot) of nAChR models on the test set. | S11 |
| **10.** | Figure S3: The statistical performance (ROC plot) of nAChR models on the test set. | S12 |
| **11.** | Figure S4: The statistical performance (ROC plot) of BuChE models on the test set. | S13 |
| **12.** | Figure S5: The statistical performance (ROC plot) of AChE models on the test set. | S14 |
| **13.** | Figure S6: The statistical performance (ROC plot) of VAChT models on the test set. | S15 |
| **14.** | Figure S7: Principle Component Analysis of Cholinergic Models (a) nAChR; (b) mAChR; (c) BuChE; (d) AChE; (e) VAChT. | S16-S20 |
| **15.** | Figure S8: The workflow to manipulate the prediction results of CWA and NPS. | S21 |
| **16.** | Table S7: The ensemble-AUC based on different machine learning method for different models. | S22 |
| **17.** | Figure S9: A typical convolutional neural network (CNN) model architecture. | S23 |
| **18.** | Table S8: The statistical performance of (CNN) model under different Array Shapes. | S24 |

**Table S1:** Dataset Description.

| **Targets** | **Collected Data** | **Training Set** | | **Test Set** | | **Total** |
| --- | --- | --- | --- | --- | --- | --- |
|  |  | **Active** | **Inactive** | **Active** | **Inactive** |  |
| nAChR | 3175 | 637 | 637 | 272 | 272 | 1818 |
| mAChR | 24223 | 2431 | 2431 | 1041 | 1041 | 6944 |
| BuChE | 2414 | 484 | 484 | 207 | 207 | 1382 |
| AChE | 8212 | 1086 | 1086 | 464 | 464 | 3098 |
| VAChT | 231 | 106 | 106 | 45 | 45 | 302 |
| CWA | 95 | - | - | - | - | 95 |
| NPS | 3126 | - | - | - | - | 3126 |

**Note: Collected Data:** For each targets, respective number of chemicals were collected from ChEMBL database, which were arranged in four activity threshold of active, inactive, margin and unclear. Among all the activity threshold, the chemicals with threshold of < 1000 nM assigned as active. Notably, to keep the balance in between active and inactive class, the decoy finder^1^ was used to select the representative chemicals from dataset with activity threshold > 1000 nM as inactive class. CWA (Chemical Warfare Agents) and NPS (New Psychoactive Substances) were considered as third set, which were used to access the machine learning model performance and were obtained from [https://www.emcdda.europa.eu](https://www.emcdda.europa.eu/) and [https://www.emcdda.europa.eu](https://www.emcdda.europa.eu/); [https://nps-datahub.com](https://nps-datahub.com/) respectively. The training and test sets contained 70% and 30% of chemicals per targets.

**Reference:**

1. Cereto-Massagué, A.; Guasch, L.; Valls, C.; Mulero, M.; Pujadas, G.; Garcia-Vallvé, S. DecoyFinder: an easy-to-use python GUI application for building target-specific decoy sets, Bioinformatics, 2012 28(12):1661-2. doi: 10.1093/bioinformatics/bts249

**Figure S1:** The workflow to build SAR classification model.


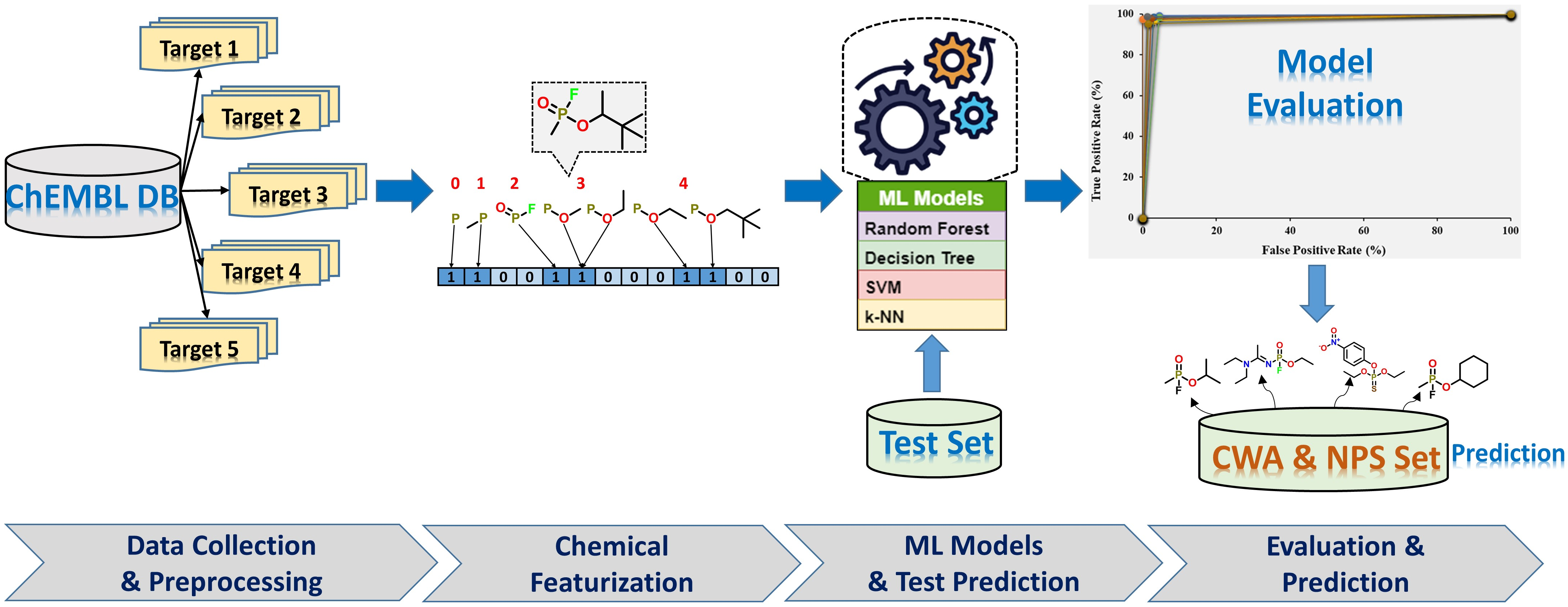


**Note:** The ChEMBL data with prerequisite target information were selected to retrieve the chemical data, which further processed for any duplicates. All the chemical data was featurized based on ECFP and FCFP (RDKit) fingerprint. The obtained feature matrix along with class data, were used to build various machine learning models, followed by the evaluation on test set. The best model was used to predict the CWA and NPS set as third sets.

**Table S2:** The statistical performance of nAChR (Nicotinic Acetylcholinesterase Receptor).

| **ML**  **Methods** | **Models** | **Train** | | | | **Test** | | | |
| --- | --- | --- | --- | --- | --- | --- | --- | --- | --- |
|  |  | **MCC** | **ACC** | **ROC** | **F-Score** | **MCC** | **ACC** | **AUC** | **F-Score** |
| **RF** | **M-1** | 0.907 | 0.954 | 0.993 | 0.954 | 0.942 | 0.971 | 0.971 | 0.971 |
|  | **M-2** | 0.918 | 0.959 | 0.994 | 0.959 | 0.975 | 0.987 | 0.987 | 0.987 |
|  | **M-3** | 0.938 | 0.969 | 0.995 | 0.969 | 0.941 | 0.971 | 0.971 | 0.970 |
|  | **M-4** | 0.934 | 0.967 | 0.994 | 0.967 | 0.938 | 0.969 | 0.969 | 0.969 |
|  | **M-5** | 0.955 | 0.977 | 0.998 | 0.977 | 0.949 | 0.974 | 0.974 | 0.974 |
|  | **M-6** | 0.947 | 0.973 | 0.997 | 0.973 | 0.942 | 0.971 | 0.971 | 0.970 |
|  | **M-7** | 0.933 | 0.966 | 0.996 | 0.966 | 0.956 | 0.978 | 0.978 | 0.978 |
|  | **M-8** | 0.950 | 0.975 | 0.997 | 0.975 | 0.956 | 0.978 | 0.978 | 0.978 |
|  | **M-9** | 0.956 | 0.978 | 0.996 | 0.978 | 0.978 | 0.989 | 0.989 | 0.989 |
|  | **M-10** | 0.922 | 0.961 | 0.993 | 0.961 | 0.938 | 0.969 | 0.969 | 0.969 |
| **DT** | **M-1** | 0.549 | 0.773 | 0.790 | 0.784 | 0.610 | 0.805 | 0.805 | 0.806 |
|  | **M-2** | 0.678 | 0.836 | 0.845 | 0.824 | 0.764 | 0.871 | 0.871 | 0.854 |
|  | **M-3** | 0.724 | 0.861 | 0.889 | 0.866 | 0.648 | 0.816 | 0.816 | 0.834 |
|  | **M-4** | 0.538 | 0.766 | 0.775 | 0.782 | 0.529 | 0.763 | 0.763 | 0.775 |
|  | **M-5** | 0.477 | 0.739 | 0.739 | 0.735 | 0.472 | 0.735 | 0.735 | 0.724 |
|  | **M-6** | 0.598 | 0.788 | 0.824 | 0.755 | 0.560 | 0.763 | 0.763 | 0.714 |
|  | **M-7** | 0.546 | 0.773 | 0.784 | 0.774 | 0.438 | 0.719 | 0.719 | 0.711 |
|  | **M-8** | 0.565 | 0.779 | 0.809 | 0.762 | 0.578 | 0.774 | 0.774 | 0.731 |
|  | **M-9** | 0.474 | 0.736 | 0.767 | 0.724 | 0.558 | 0.759 | 0.759 | 0.704 |
|  | **M-10** | 0.657 | 0.827 | 0.843 | 0.820 | 0.666 | 0.825 | 0.825 | 0.805 |
| **SVM** | **M-1** | 0.934 | 0.967 | 0.989 | 0.967 | 0.956 | 0.978 | 0.978 | 0.978 |
|  | **M-2** | 0.936 | 0.968 | 0.994 | 0.968 | 0.978 | 0.989 | 0.989 | 0.989 |
|  | **M-3** | 0.955 | 0.977 | 0.997 | 0.977 | 0.953 | 0.976 | 0.976 | 0.976 |
|  | **M-4** | 0.937 | 0.969 | 0.995 | 0.969 | 0.938 | 0.969 | 0.969 | 0.969 |
|  | **M-5** | 0.950 | 0.975 | 0.997 | 0.975 | 0.945 | 0.972 | 0.972 | 0.972 |
|  | **M-6** | 0.934 | 0.967 | 0.993 | 0.967 | 0.945 | 0.972 | 0.972 | 0.972 |
|  | **M-7** | 0.939 | 0.969 | 0.996 | 0.969 | 0.949 | 0.974 | 0.974 | 0.975 |
|  | **M-8** | 0.936 | 0.968 | 0.995 | 0.968 | 0.964 | 0.982 | 0.982 | 0.982 |
|  | **M-9** | 0.939 | 0.969 | 0.989 | 0.969 | 0.952 | 0.976 | 0.976 | 0.976 |
|  | **M-10** | 0.934 | 0.967 | 0.991 | 0.967 | 0.960 | 0.980 | 0.980 | 0.980 |
| **KNN** | **M-1** | 0.778 | 0.887 | 0.972 | 0.892 | 0.791 | 0.892 | 0.892 | 0.899 |
|  | **M-2** | 0.551 | 0.741 | 0.965 | 0.791 | 0.558 | 0.737 | 0.737 | 0.792 |
|  | **M-3** | 0.722 | 0.849 | 0.975 | 0.866 | 0.729 | 0.853 | 0.853 | 0.869 |
|  | **M-4** | 0.506 | 0.704 | 0.961 | 0.772 | 0.475 | 0.684 | 0.684 | 0.760 |
|  | **M-5** | 0.693 | 0.827 | 0.983 | 0.851 | 0.723 | 0.846 | 0.972 | 0.865 |
|  | **M-6** | 0.675 | 0.814 | 0.975 | 0.843 | 0.674 | 0.816 | 0.816 | 0.843 |
|  | **M-7** | 0.619 | 0.779 | 0.967 | 0.818 | 0.646 | 0.796 | 0.796 | 0.830 |
|  | **M-8** | 0.638 | 0.790 | 0.980 | 0.826 | 0.647 | 0.730 | 0.798 | 0.831 |
|  | **M-9** | 0.506 | 0.705 | 0.971 | 0.772 | 0.546 | 0.730 | 0.730 | 0.787 |
|  | **M-10** | 0.643 | 0.799 | 0.970 | 0.830 | 0.687 | 0.822 | 0.822 | 0.848 |

**Abbreviations:** **MCC:** Matthew’s Correlation Coefficient; **ACC:** Accuracy; **ROC:** Receiver Operative Characteristic; **F-Score:** Fisher Value; **AUC:** Area Under the Curve; **RF:** Random Forest; **DT:** Decision Tree; **SVM:** Support Vector Machine; **KNN:** K-Nearest Neighbor.

**Table S3:** The statistical performance of mAChR (Muscarinic Acetylcholinesterase Receptor).

| **ML**  **Methods** | **Models** | **Train** | | | | **Test** | | | |
| --- | --- | --- | --- | --- | --- | --- | --- | --- | --- |
|  |  | **MCC** | **ACC** | **ROC** | **F-Score** | **MCC** | **ACC** | **AUC** | **F-Score** |
| **RF** | **M-1** | 0.945 | 0.972 | 0.996 | 0.972 | 0.935 | 0.967 | 0.967 | 0.967 |
|  | **M-2** | 0.959 | 0.979 | 0.997 | 0.979 | 0.974 | 0.987 | 0.987 | 0.987 |
|  | **M-3** | 0.965 | 0.983 | 0.998 | 0.983 | 0.966 | 0.983 | 0.983 | 0.983 |
|  | **M-4** | 0.929 | 0.965 | 0.995 | 0.965 | 0.927 | 0.963 | 0.963 | 0.964 |
|  | **M-5** | 0.959 | 0.980 | 0.997 | 0.980 | 0.968 | 0.984 | 0.984 | 0.984 |
|  | **M-6** | 0.952 | 0.976 | 0.997 | 0.976 | 0.954 | 0.977 | 0.977 | 0.977 |
|  | **M-7** | 0.964 | 0.982 | 0.998 | 0.982 | 0.965 | 0.983 | 0.983 | 0.983 |
|  | **M-8** | 0.964 | 0.982 | 0.998 | 0.982 | 0.966 | 0.983 | 0.983 | 0.983 |
|  | **M-9** | 0.932 | 0.966 | 0.995 | 0.966 | 0.933 | 0.966 | 0.966 | 0.966 |
|  | **M-10** | 0.938 | 0.969 | 0.996 | 0.969 | 0.928 | 0.964 | 0.964 | 0.964 |
| **DT** | **M-1** | 0.650 | 0.825 | 0.826 | 0.823 | 0.608 | 0.804 | 0.804 | 0.799 |
|  | **M-2** | 0.663 | 0.831 | 0.837 | 0.827 | 0.681 | 0.840 | 0.840 | 0.833 |
|  | **M-3** | 0.731 | 0.865 | 0.869 | 0.860 | 0.725 | 0.860 | 0.860 | 0.852 |
|  | **M-4** | 0.693 | 0.846 | 0.858 | 0.845 | 0.665 | 0.831 | 0.831 | 0.824 |
|  | **M-5** | 0.696 | 0.847 | 0.853 | 0.843 | 0.655 | 0.826 | 0.826 | 0.817 |
|  | **M-6** | 0.673 | 0.837 | 0.841 | 0.834 | 0.642 | 0.820 | 0.820 | 0.813 |
|  | **M-7** | 0.666 | 0.832 | 0.838 | 0.833 | 0.658 | 0.828 | 0.828 | 0.821 |
|  | **M-8** | 0.711 | 0.855 | 0.861 | 0.852 | 0.701 | 0.849 | 0.849 | 0.841 |
|  | **M-9** | 0.663 | 0.832 | 0.844 | 0.831 | 0.648 | 0.822 | 0.822 | 0.812 |
|  | **M-10** | 0.599 | 0.799 | 0.807 | 0.799 | 0.615 | 0.806 | 0.806 | 0.799 |
| **SVM** | **M-1** | 0.940 | 0.970 | 0.992 | 0.970 | 0.937 | 0.968 | 0.968 | 0.968 |
|  | **M-2** | 0.962 | 0.981 | 0.997 | 0.981 | 0.967 | 0.984 | 0.984 | 0.984 |
|  | **M-3** | 0.962 | 0.981 | 0.996 | 0.981 | 0.967 | 0.984 | 0.984 | 0.984 |
|  | **M-4** | 0.938 | 0.969 | 0.992 | 0.969 | 0.936 | 0.968 | 0.968 | 0.968 |
|  | **M-5** | 0.968 | 0.984 | 0.997 | 0.984 | 0.973 | 0.987 | 0.987 | 0.987 |
|  | **M-6** | 0.959 | 0.979 | 0.996 | 0.979 | 0.962 | 0.981 | 0.981 | 0.981 |
|  | **M-7** | 0.968 | 0.984 | 0.997 | 0.984 | 0.961 | 0.980 | 0.980 | 0.980 |
|  | **M-8** | 0.968 | 0.984 | 0.998 | 0.984 | 0.965 | 0.983 | 0.983 | 0.983 |
|  | **M-9** | 0.934 | 0.967 | 0.991 | 0.967 | 0.941 | 0.970 | 0.970 | 0.970 |
|  | **M-10** | 0.928 | 0.964 | 0.992 | 0.964 | 0.940 | 0.970 | 0.970 | 0.970 |
| **KNN** | **M-1** | 0.896 | 0.948 | 0.989 | 0.948 | 0.896 | 0.948 | 0.948 | 0.948 |
|  | **M-2** | 0.876 | 0.934 | 0.987 | 0.93 | 0.895 | 0.945 | 0.945 | 0.942 |
|  | **M-3** | 0.904 | 0.950 | 0.989 | 0.947 | 0.894 | 0.945 | 0.945 | 0.942 |
|  | **M-4** | 0.888 | 0.942 | 0.985 | 0.940 | 0.916 | 0.957 | 0.957 | 0.955 |
|  | **M-5** | 0.868 | 0.930 | 0.985 | 0.925 | 0.873 | 0.932 | 0.932 | 0.927 |
|  | **M-6** | 0.911 | 0.956 | 0.992 | 0.955 | 0.917 | 0.958 | 0.958 | 0.958 |
|  | **M-7** | 0.917 | 0.958 | 0.993 | 0.956 | 0.932 | 0.965 | 0.965 | 0.965 |
|  | **M-8** | 0.898 | 0.947 | 0.989 | 0.944 | 0.899 | 0.947 | 0.947 | 0.944 |
|  | **M-9** | 0.883 | 0.939 | 0.982 | 0.936 | 0.888 | 0.942 | 0.942 | 0.939 |
|  | **M-10** | 0.862 | 0.930 | 0.987 | 0.932 | 0.839 | 0.919 | 0.919 | 0.921 |

**Abbreviations:** **MCC:** Matthew’s Correlation Coefficient; **ACC:** Accuracy; **ROC:** Receiver Operative Characteristic; **F-Score:** Fisher Value; **AUC:** Area Under the Curve; **RF:** Random Forest; **DT:** Decision Tree; **SVM:** Support Vector Machine; **KNN:** K-Nearest Neighbor.

**Table S4:** The statistical performance of BuChE (Butyryl cholinesterase Enzyme).

| **ML**  **Methods** | **Models** | **Train** | | | | **Test** | | | |
| --- | --- | --- | --- | --- | --- | --- | --- | --- | --- |
|  |  | **MCC** | **ACC** | **ROC** | **F-Score** | **MCC** | **ACC** | **AUC** | **F-Score** |
| **RF** | **M-1** | 0.949 | 0.974 | 0.999 | 0.974 | 0.948 | 0.973 | 0.973 | 0.973 |
|  | **M-2** | 0.969 | 0.985 | 0.999 | 0.985 | 0.971 | 0.986 | 0.986 | 0.985 |
|  | **M-3** | 0.977 | 0.989 | 0.999 | 0.989 | 0.952 | 0.976 | 0.976 | 0.976 |
|  | **M-4** | 0.971 | 0.986 | 0.999 | 0.986 | 0.986 | 0.993 | 0.993 | 0.993 |
|  | **M-5** | 0.953 | 0.976 | 0.997 | 0.976 | 0.943 | 0.971 | 0.971 | 0.970 |
|  | **M-6** | 0.953 | 0.976 | 0.996 | 0.976 | 0.957 | 0.978 | 0.978 | 0.978 |
|  | **M-7** | 0.933 | 0.966 | 0.992 | 0.965 | 0.948 | 0.973 | 0.973 | 0.973 |
|  | **M-8** | 0.949 | 0.974 | 0.995 | 0.974 | 0.948 | 0.973 | 0.973 | 0.973 |
|  | **M-9** | 0.938 | 0.969 | 0.994 | 0.969 | 0.952 | 0.976 | 0.976 | 0.976 |
|  | **M-10** | 0.973 | 0.987 | 0.999 | 0.987 | 0.933 | 0.966 | 0.966 | 0.967 |
| **DT** | **M-1** | 0.523 | 0.761 | 0.796 | 0.760 | 0.566 | 0.773 | 0.773 | 0.799 |
|  | **M-2** | 0.655 | 0.826 | 0.864 | 0.833 | 0.644 | 0.821 | 0.821 | 0.816 |
|  | **M-3** | 0.660 | 0.830 | 0.892 | 0.833 | 0.649 | 0.821 | 0.821 | 0.833 |
|  | **M-4** | 0.699 | 0.848 | 0.874 | 0.854 | 0.567 | 0.783 | 0.783 | 0.791 |
|  | **M-5** | 0.641 | 0.810 | 0.818 | 0.782 | 0.604 | 0.780 | 0.780 | 0.730 |
|  | **M-6** | 0.647 | 0.807 | 0.809 | 0.771 | 0.674 | 0.814 | 0.814 | 0.773 |
|  | **M-7** | 0.574 | 0.775 | 0.780 | 0.737 | 0.522 | 0.754 | 0.754 | 0.721 |
|  | **M-8** | 0.557 | 0.770 | 0.771 | 0.737 | 0.535 | 0.754 | 0.754 | 0.707 |
|  | **M-9** | 0.660 | 0.816 | 0.819 | 0.786 | 0.592 | 0.780 | 0.780 | 0.738 |
|  | **M-10** | 0.807 | 0.902 | 0.912 | 0.906 | 0.763 | 0.874 | 0.874 | 0.886 |
| **SVM** | **M-1** | 0.961 | 0.980 | 0.995 | 0.980 | 0.947 | 0.973 | 0.973 | 0.973 |
|  | **M-2** | 0.971 | 0.986 | 0.999 | 0.985 | 0.986 | 0.993 | 0.993 | 0.993 |
|  | **M-3** | 0.973 | 0.987 | 0.942 | 0.987 | 0.952 | 0.976 | 0.976 | 0.975 |
|  | **M-4** | 0.957 | 0.978 | 0.998 | 0.978 | 0.976 | 0.988 | 0.988 | 0.988 |
|  | **M-5** | 0.969 | 0.985 | 0.997 | 0.985 | 0.947 | 0.973 | 0.973 | 0.973 |
|  | **M-6** | 0.959 | 0.979 | 0.997 | 0.979 | 0.981 | 0.990 | 0.990 | 0.990 |
|  | **M-7** | 0.945 | 0.972 | 0.992 | 0.972 | 0.952 | 0.976 | 0.976 | 0.976 |
|  | **M-8** | 0.958 | 0.979 | 0.997 | 0.979 | 0.947 | 0.973 | 0.973 | 0.973 |
|  | **M-9** | 0.946 | 0.973 | 0.993 | 0.973 | 0.961 | 0.981 | 0.981 | 0.981 |
|  | **M-10** | 0.981 | 0.991 | 1.000 | 0.991 | 0.937 | 0.969 | 0.969 | 0.969 |
| **KNN** | **M-1** | 0.408 | 0.643 | 0.909 | 0.737 | 0.447 | 0.667 | 0.667 | 0.750 |
|  | **M-2** | 0.404 | 0.641 | 0.921 | 0.736 | 0.451 | 0.669 | 0.669 | 0.751 |
|  | **M-3** | 0.420 | 0.741 | 1.000 | 0.682 | 0.583 | 0.754 | 0.754 | 0.802 |
|  | **M-4** | 0.558 | 0.738 | 0.963 | 0.792 | 0.620 | 0.778 | 0.778 | 0.818 |
|  | **M-5** | 0.421 | 0.651 | 0.924 | 0.741 | 0.420 | 0.650 | 0.650 | 0.741 |
|  | **M-6** | 0.606 | 0.769 | 0.938 | 0.812 | 0.650 | 0.797 | 0.797 | 0.831 |
|  | **M-7** | 0.397 | 0.636 | 0.885 | 0.733 | 0.459 | 0.674 | 0.674 | 0.754 |
|  | **M-8** | 0.455 | 0.672 | 0.902 | 0.753 | 0.459 | 0.674 | 0.674 | 0.754 |
|  | **M-9** | 0.569 | 0.745 | 0.944 | 0.797 | 0.587 | 0.756 | 0.756 | 0.804 |
|  | **M-10** | 0.584 | 0.754 | 0.985 | 0.803 | 0.620 | 0.778 | 0.778 | 0.818 |

**Abbreviations:** **MCC:** Matthew’s Correlation Coefficient; **ACC:** Accuracy; **ROC:** Receiver Operative Characteristic; **F-Score:** Fisher Value; **AUC:** Area Under the Curve; **RF:** Random Forest; **DT:** Decision Tree; **SVM:** Support Vector Machine; **KNN:** K-Nearest Neighbor.

**Table S5:** The statistical performance of AChE (Acetylcholinesterase Enzyme).

| **ML**  **Methods** | **Models** | **Train** | | | | **Test** | | | |
| --- | --- | --- | --- | --- | --- | --- | --- | --- | --- |
|  |  | **MCC** | **ACC** | **ROC** | **F-Score** | **MCC** | **ACC** | **AUC** | **F-Score** |
| **RF** | **M-1** | 0.940 | 0.970 | 0.995 | 0.969 | 0.951 | 0.975 | 0.975 | 0.975 |
|  | **M-2** | 0.965 | 0.983 | 0.999 | 0.983 | 0.963 | 0.982 | 0.982 | 0.982 |
|  | **M-3** | 0.942 | 0.971 | 0.997 | 0.971 | 0.962 | 0.981 | 0.981 | 0.981 |
|  | **M-4** | 0.936 | 0.968 | 0.994 | 0.967 | 0.959 | 0.980 | 0.980 | 0.979 |
|  | **M-5** | 0.934 | 0.967 | 0.992 | 0.966 | 0.910 | 0.955 | 0.955 | 0.954 |
|  | **M-6** | 0.933 | 0.966 | 0.994 | 0.966 | 0.929 | 0.964 | 0.964 | 0.964 |
|  | **M-7** | 0.917 | 0.959 | 0.994 | 0.958 | 0.953 | 0.976 | 0.976 | 0.976 |
|  | **M-8** | 0.923 | 0.961 | 0.991 | 0.961 | 0.944 | 0.972 | 0.972 | 0.972 |
|  | **M-9** | 0.929 | 0.964 | 0.992 | 0.964 | 0.931 | 0.966 | 0.966 | 0.965 |
|  | **M-10** | 0.948 | 0.974 | 0.998 | 0.974 | 0.944 | 0.972 | 0.972 | 0.972 |
| **DT** | **M-1** | 0.542 | 0.771 | 0.785 | 0.776 | 0.514 | 0.755 | 0.755 | 0.768 |
|  | **M-2** | 0.570 | 0.779 | 0.806 | 0.755 | 0.586 | 0.778 | 0.778 | 0.737 |
|  | **M-3** | 0.627 | 0.808 | 0.832 | 0.824 | 0.597 | 0.789 | 0.789 | 0.813 |
|  | **M-4** | 0.652 | 0.813 | 0.817 | 0.783 | 0.678 | 0.828 | 0.828 | 0.802 |
|  | **M-5** | 0.648 | 0.816 | 0.821 | 0.794 | 0.609 | 0.796 | 0.796 | 0.770 |
|  | **M-6** | 0.624 | 0.806 | 0.812 | 0.784 | 0.597 | 0.789 | 0.789 | 0.758 |
|  | **M-7** | 0.537 | 0.769 | 0.774 | 0.770 | 0.507 | 0.753 | 0.753 | 0.746 |
|  | **M-8** | 0.666 | 0.824 | 0.830 | 0.802 | 0.637 | 0.803 | 0.803 | 0.767 |
|  | **M-9** | 0.674 | 0.825 | 0.829 | 0.799 | 0.644 | 0.806 | 0.806 | 0.770 |
|  | **M-10** | 0.620 | 0.794 | 0.818 | 0.822 | 0.653 | 0.801 | 0.801 | 0.833 |
| **SVM** | **M-1** | 0.936 | 0.968 | 0.994 | 0.967 | 0.940 | 0.970 | 0.970 | 0.970 |
|  | **M-2** | 0.979 | 0.989 | 0.999 | 0.989 | 0.974 | 0.987 | 0.987 | 0.987 |
|  | **M-3** | 0.943 | 0.971 | 0.996 | 0.972 | 0.972 | 0.986 | 0.986 | 0.986 |
|  | **M-4** | 0.929 | 0.965 | 0.993 | 0.964 | 0.933 | 0.967 | 0.967 | 0.967 |
|  | **M-5** | 0.926 | 0.963 | 0.990 | 0.963 | 0.912 | 0.956 | 0.956 | 0.956 |
|  | **M-6** | 0.923 | 0.961 | 0.988 | 0.962 | 0.912 | 0.956 | 0.956 | 0.956 |
|  | **M-7** | 0.915 | 0.958 | 0.989 | 0.958 | 0.925 | 0.962 | 0.962 | 0.963 |
|  | **M-8** | 0.932 | 0.966 | 0.994 | 0.966 | 0.881 | 0.954 | 0.969 | 0.969 |
|  | **M-9** | 0.919 | 0.959 | 0.988 | 0.959 | 0.925 | 0.962 | 0.962 | 0.962 |
|  | **M-10** | 0.946 | 0.973 | 0.996 | 0.973 | 0.957 | 0.978 | 0.978 | 0.978 |
| **KNN** | **M-1** | 0.614 | 0.774 | 0.983 | 0.816 | 0.586 | 0.757 | 0.756 | 0.804 |
|  | **M-2** | 0.715 | 0.838 | 0.968 | 0.861 | 0.722 | 0.842 | 0.844 | 0.863 |
|  | **M-3** | 0.704 | 0.832 | 0.982 | 0.856 | 0.683 | 0.818 | 0.818 | 0.846 |
|  | **M-4** | 0.879 | 0.939 | 0.983 | 0.939 | 0.871 | 0.935 | 0.935 | 0.936 |
|  | **M-5** | 0.500 | 0.702 | 0.977 | 0.770 | 0.481 | 0.691 | 0.691 | 0.763 |
|  | **M-6** | 0.448 | 0.668 | 0.922 | 0.750 | 0.434 | 0.658 | 0.658 | 0.745 |
|  | **M-7** | 0.383 | 0.629 | 0.855 | 0.729 | 0.397 | 0.638 | 0.638 | 0.734 |
|  | **M-8** | 0.520 | 0.713 | 0.958 | 0.777 | 0.533 | 0.721 | 0.721 | 0.782 |
|  | **M-9** | 0.612 | 0.779 | 0.963 | 0.817 | 0.592 | 0.767 | 0.767 | 0.809 |
|  | **M-10** | 0.701 | 0.830 | 0.985 | 0.855 | 0.721 | 0.843 | 0.843 | 0.864 |

**Abbreviations:** **MCC:** Matthew’s Correlation Coefficient; **ACC:** Accuracy; **ROC:** Receiver Operative Characteristic; **F-Score:** Fisher Value; **AUC:** Area Under the Curve; **RF:** Random Forest; **DT:** Decision Tree; **SVM:** Support Vector Machine; **KNN:** K-Nearest Neighbor.

**Table S6:** The statistical performance of VAChT (Vesicular Acetylcholine Transporter).

| **ML**  **Methods** | **Models** | **Train** | | | | **Test** | | | |
| --- | --- | --- | --- | --- | --- | --- | --- | --- | --- |
|  |  | **MCC** | **ACC** | **ROC** | **F-Score** | **MCC** | **ACC** | **AUC** | **F-Score** |
| **RF** | **M-1** | 0.658 | 0.802 | 0.995 | 0.835 | 0.620 | 0.778 | 0.778 | 0.818 |
|  | **M-2** | 0.563 | 0.741 | 1.000 | 0.794 | 0.429 | 0.656 | 0.656 | 0.744 |
|  | **M-3** | 0.991 | 0.995 | 1.000 | 0.995 | 1.000 | 1.000 | 0.778 | 1.000 |
|  | **M-4** | 0.771 | 0.873 | 1.000 | 0.887 | 0.603 | 0.767 | 0.767 | 0.811 |
|  | **M-5** | 0.702 | 0.830 | 1.000 | 0.887 | 0.915 | 0.956 | 0.911 | 0.957 |
|  | **M-6** | 0.702 | 0.830 | 1.000 | 0.855 | 0.290 | 0.578 | 0.578 | 0.703 |
|  | **M-7** | 0.972 | 0.986 | 1.000 | 0.986 | 0.978 | 0.989 | 0.989 | 0.988 |
|  | **M-8** | 0.815 | 0.901 | 0.998 | 0.909 | 0.875 | 0.933 | 0.933 | 0.938 |
|  | **M-9** | 0.981 | 0.991 | 1.000 | 0.991 | 0.978 | 0.989 | 0.989 | 0.989 |
|  | **M-10** | 0.981 | 0.991 | 1.000 | 0.991 | 1.000 | 1.000 | 1.000 | 1.000 |
| **DT** | **M-1** | 0.906 | 0.953 | 0.956 | 0.954 | 0.891 | 0.944 | 0.944 | 0.946 |
|  | **M-2** | 0.815 | 0.906 | 0.945 | 0.910 | 0.800 | 0.900 | 0.900 | 0.899 |
|  | **M-3** | 0.944 | 0.972 | 0.970 | 0.972 | 0.915 | 0.956 | 0.944 | 0.957 |
|  | **M-4** | 0.887 | 0.943 | 0.971 | 0.943 | 0.850 | 0.922 | 0.922 | 0.926 |
|  | **M-5** | 0.953 | 0.976 | 0.975 | 0.976 | 0.934 | 0.967 | 0.944 | 0.966 |
|  | **M-6** | 0.962 | 0.981 | 0.981 | 0.981 | 0.889 | 0.944 | 0.944 | 0.945 |
|  | **M-7** | 0.877 | 0.939 | 0.958 | 0.938 | 0.911 | 0.956 | 0.956 | 0.955 |
|  | **M-8** | 0.953 | 0.976 | 0.976 | 0.977 | 0.912 | 0.956 | 0.956 | 0.957 |
|  | **M-9** | 0.962 | 0.981 | 0.981 | 0.981 | 0.957 | 0.978 | 0.978 | 0.978 |
|  | **M-10** | 0.953 | 0.976 | 0.976 | 0.977 | 0.957 | 0.978 | 0.989 | 0.978 |
| **SVM** | **M-1** | 0.953 | 0.976 | 0.993 | 0.976 | 1.000 | 1.000 | 1.000 | 1.000 |
|  | **M-2** | 0.991 | 0.995 | 1.000 | 0.995 | 0.957 | 0.978 | 0.978 | 0.977 |
|  | **M-3** | 1.000 | 1.000 | 1.000 | 1.000 | 1.000 | 1.000 | 1.000 | 1.000 |
|  | **M-4** | 0.981 | 0.991 | 0.998 | 0.991 | 1.000 | 1.000 | 1.000 | 1.000 |
|  | **M-5** | 0.991 | 0.995 | 0.998 | 0.991 | 1.000 | 1.000 | 1.000 | 1.000 |
|  | **M-6** | 0.981 | 0.991 | 0.998 | 0.991 | 1.000 | 1.000 | 1.000 | 1.000 |
|  | **M-7** | 0.991 | 0.995 | 0.999 | 0.995 | 1.000 | 1.000 | 1.000 | 1.000 |
|  | **M-8** | 0.991 | 0.995 | 0.999 | 0.995 | 1.000 | 1.000 | 1.000 | 1.000 |
|  | **M-9** | 1.000 | 1.000 | 1.000 | 1.000 | 0.978 | 0.989 | 0.989 | 0.989 |
|  | **M-10** | 0.972 | 0.986 | 0.998 | 0.986 | 1.000 | 1.000 | 1.000 | 1.000 |
| **KNN** | **M-1** | 0.429 | 0.656 | 0.846 | 0.744 | 0.373 | 0.622 | 0.622 | 0.726 |
|  | **M-2** | 0.357 | 0.613 | 0.879 | 0.721 | 0.392 | 0.633 | 0.633 | 0.732 |
|  | **M-3** | 0.925 | 0.962 | 0.996 | 0.963 | 0.957 | 0.978 | 0.622 | 0.978 |
|  | **M-4** | 0.944 | 0.972 | 0.996 | 0.972 | 0.934 | 0.967 | 0.967 | 0.967 |
|  | **M-5** | 0.953 | 0.976 | 0.998 | 0.977 | 0.934 | 0.967 | 0.956 | 0.967 |
|  | **M-6** | 0.991 | 0.995 | 1.000 | 0.995 | 0.957 | 0.957 | 0.978 | 0.978 |
|  | **M-7** | 0.991 | 0.995 | 0.995 | 0.995 | 0.978 | 0.989 | 0.989 | 0.989 |
|  | **M-8** | 0.981 | 0.991 | 0.995 | 0.991 | 0.978 | 0.989 | 0.989 | 0.989 |
|  | **M-9** | 0.925 | 0.962 | 0.999 | 0.963 | 0.915 | 0.956 | 0.956 | 0.957 |
|  | **M-10** | 0.934 | 0.967 | 1.000 | 0.967 | 0.957 | 0.978 | 0.989 | 0.978 |

**Abbreviations:** **MCC:** Matthew’s Correlation Coefficient; **ACC:** Accuracy; **ROC:** Receiver Operative Characteristic; **F-Score:** Fisher Value; **AUC:** Area Under the Curve; **RF:** Random Forest; **DT:** Decision Tree; **SVM:** Support Vector Machine; **KNN:** K-Nearest Neighbor.

**Table S7:** The ensemble-AUC based on different machine learning method for different models.

| **Models** | **Ensemble-AUC** | | | | |
| --- | --- | --- | --- | --- | --- |
|  | **nAChR** | **mAChR** | **AChE** | **BuChE** | **VAChT** |
| **M-1** | 0.708 | 0.378 | 0.495 | **0.475** | 0.732 |
| **M-2** | **0.749** | 0.370 | 0.502 | 0.443 | 0.739 |
| **M-3** | 0.729 | 0.370 | **0.517** | 0.459 | 0.729 |
| **M-4** | 0.720 | 0.357 | 0.502 | 0.470 | 0.778 |
| **M-5** | 0.714 | 0.370 | 0.499 | 0.457 | **0.790** |
| **M-6** | 0.721 | **0.380** | 0.506 | 0.449 | 0.753 |
| **M-7** | 0.718 | 0.370 | 0.500 | 0.460 | 0.775 |
| **M-8** | 0.180 | 0.364 | 0.510 | 0.467 | 0.752 |
| **M-9** | 0.709 | 0.364 | 0.498 | 0.441 | 0.709 |
| **M-10** | 0.712 | 0.377 | 0.500 | 0.452 | 0.786 |

**Abbreviations:** **nAChR:** Nicotinic Acetylcholinesterase Receptor; **mAChR:** Muscarinic Acetylcholinesterase Receptor; **AChE:** Acetylcholinesterase Enzyme; **BuChE:** Butyryl cholinesterase Enzyme; **VAChT:** Vesicular Acetylcholine Transporter.

**Figure S2:** The statistical performance (ROC plot) of nAChR models on the test set.


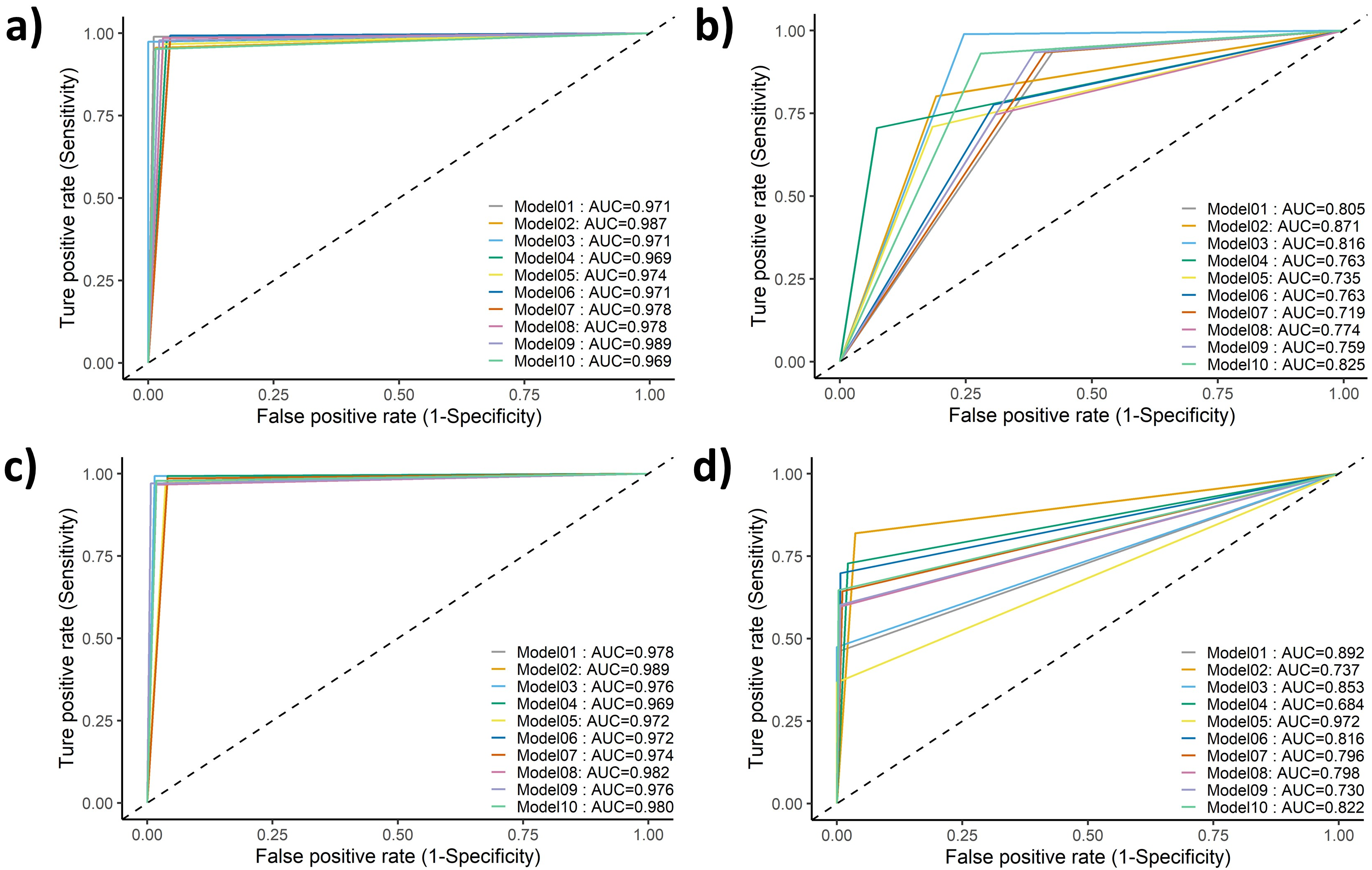


**Figure S3:** The statistical performance (ROC plot) of mAChR models on the test set.


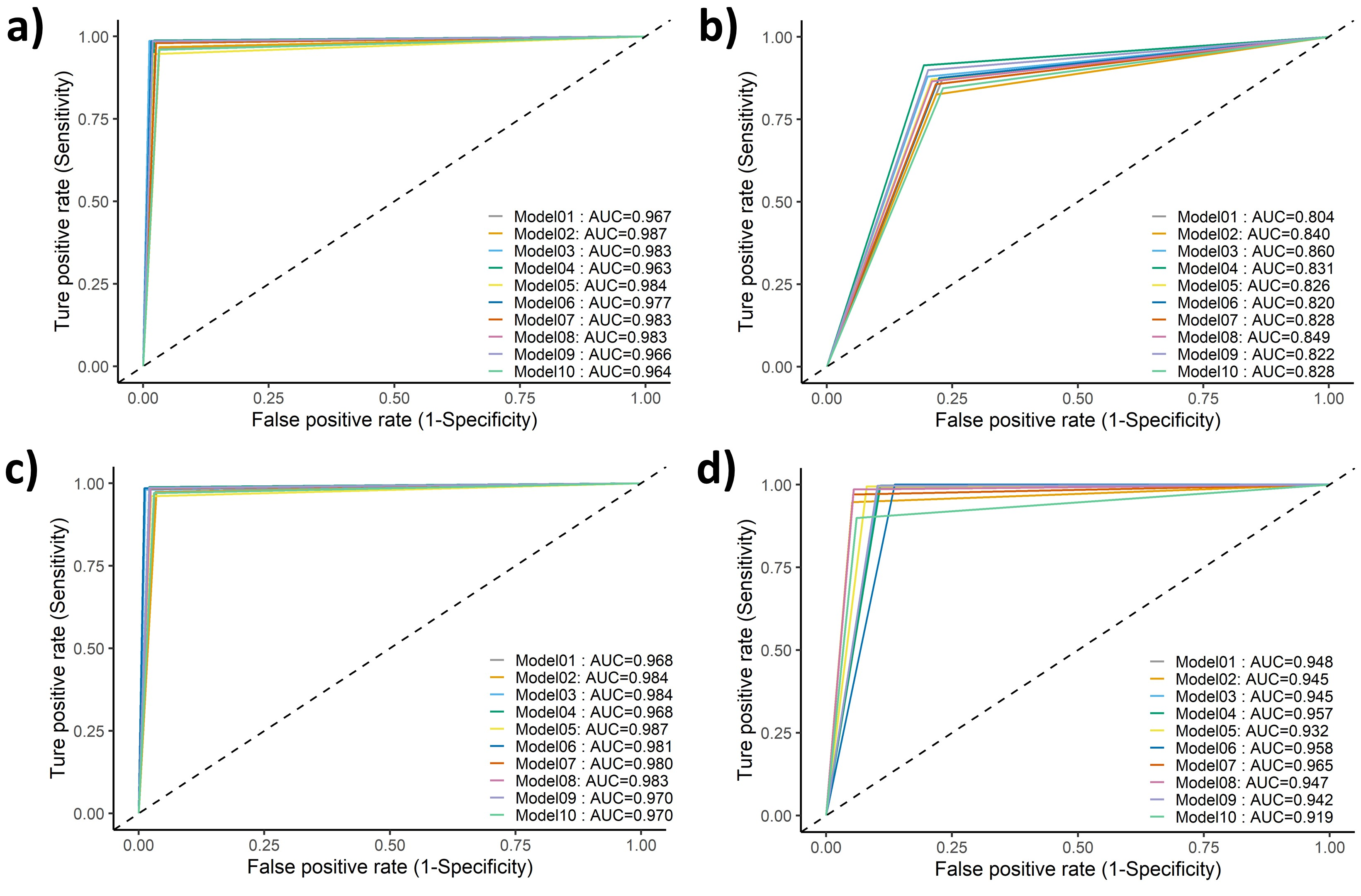


**Figure S4:** The statistical performance (ROC plot) of BuChE models on the test set.


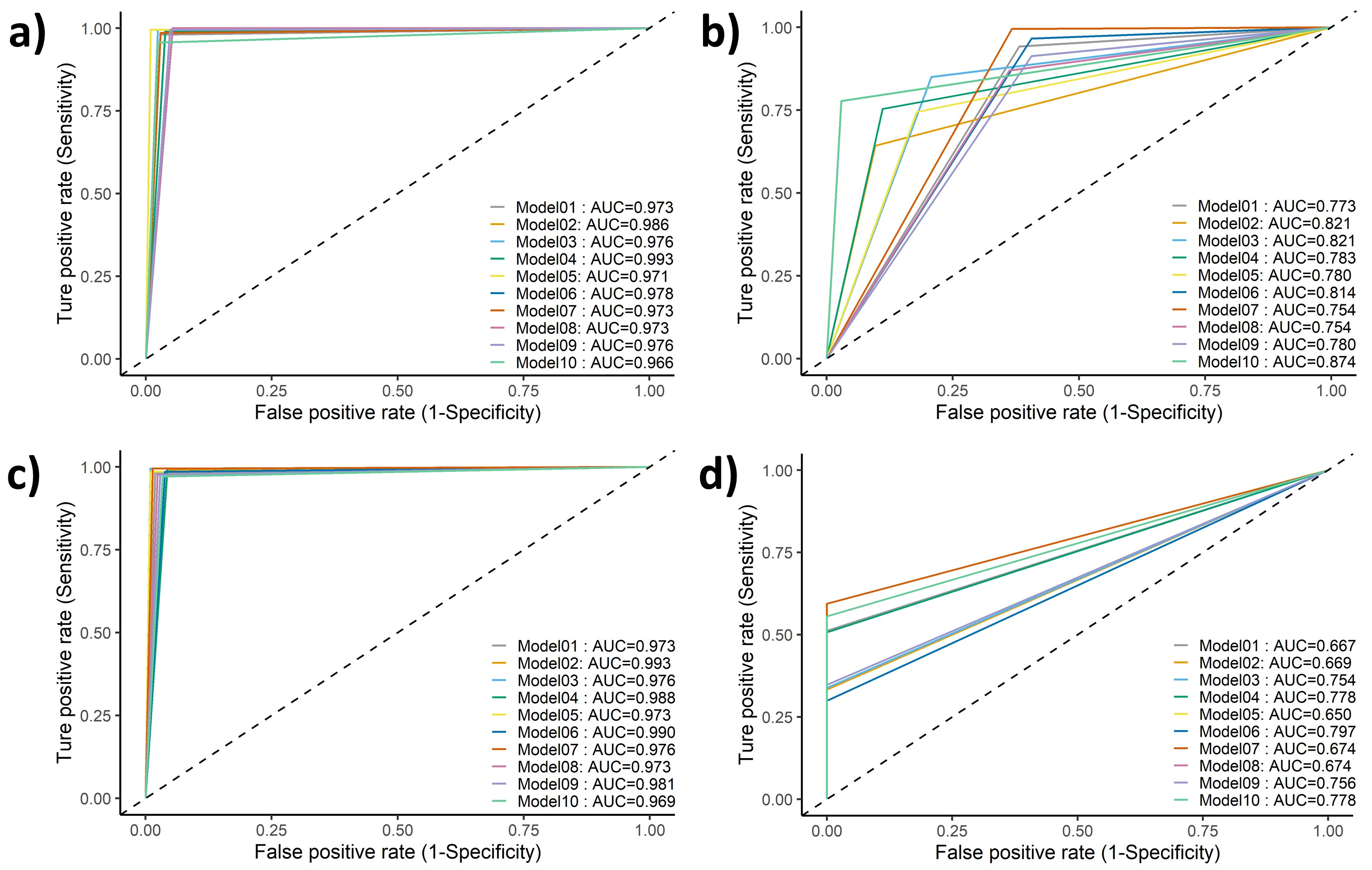


**Figure S5:** The statistical performance (ROC plot) of AChE models on the test set.


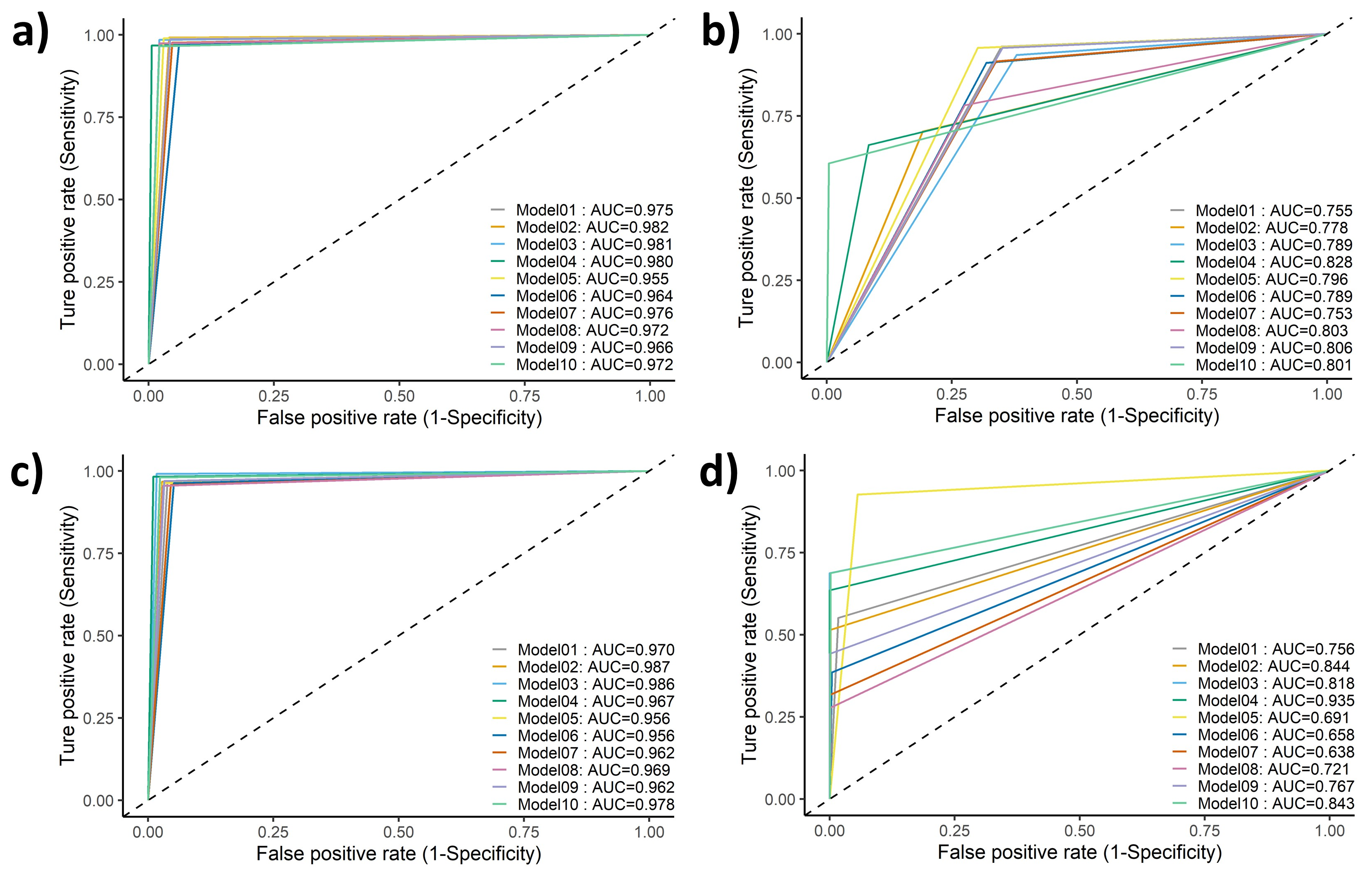


**Figure S6:** The statistical performance (ROC plot) of VAChT models on the test set.


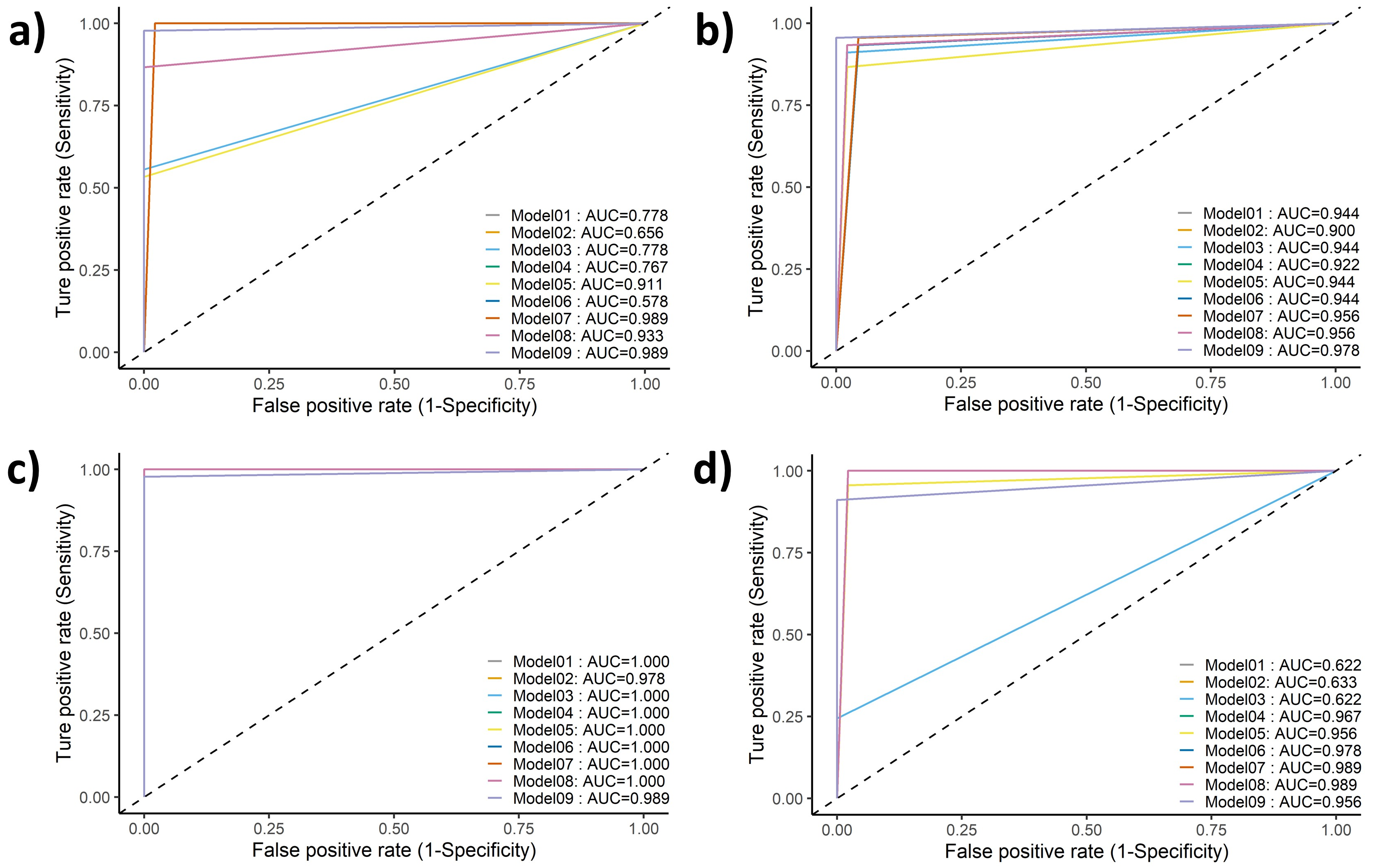


**Figure S7:** Principle Component Analysis of Cholinergic Models (a) nAChR; (b) mAChR; (c) BuChE; (d) AChE; (e) VAChT.

**(a)**


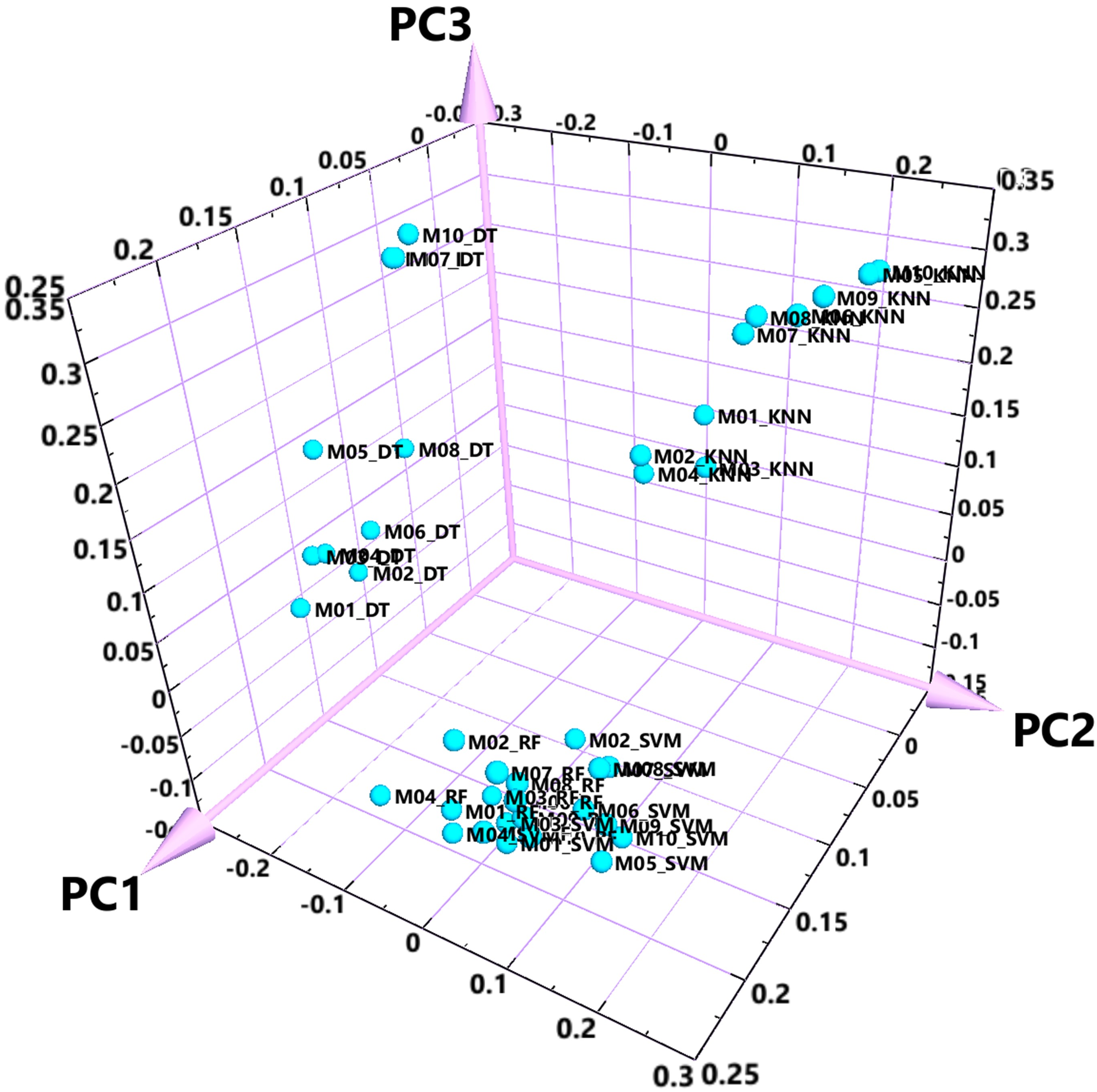


**(b)**


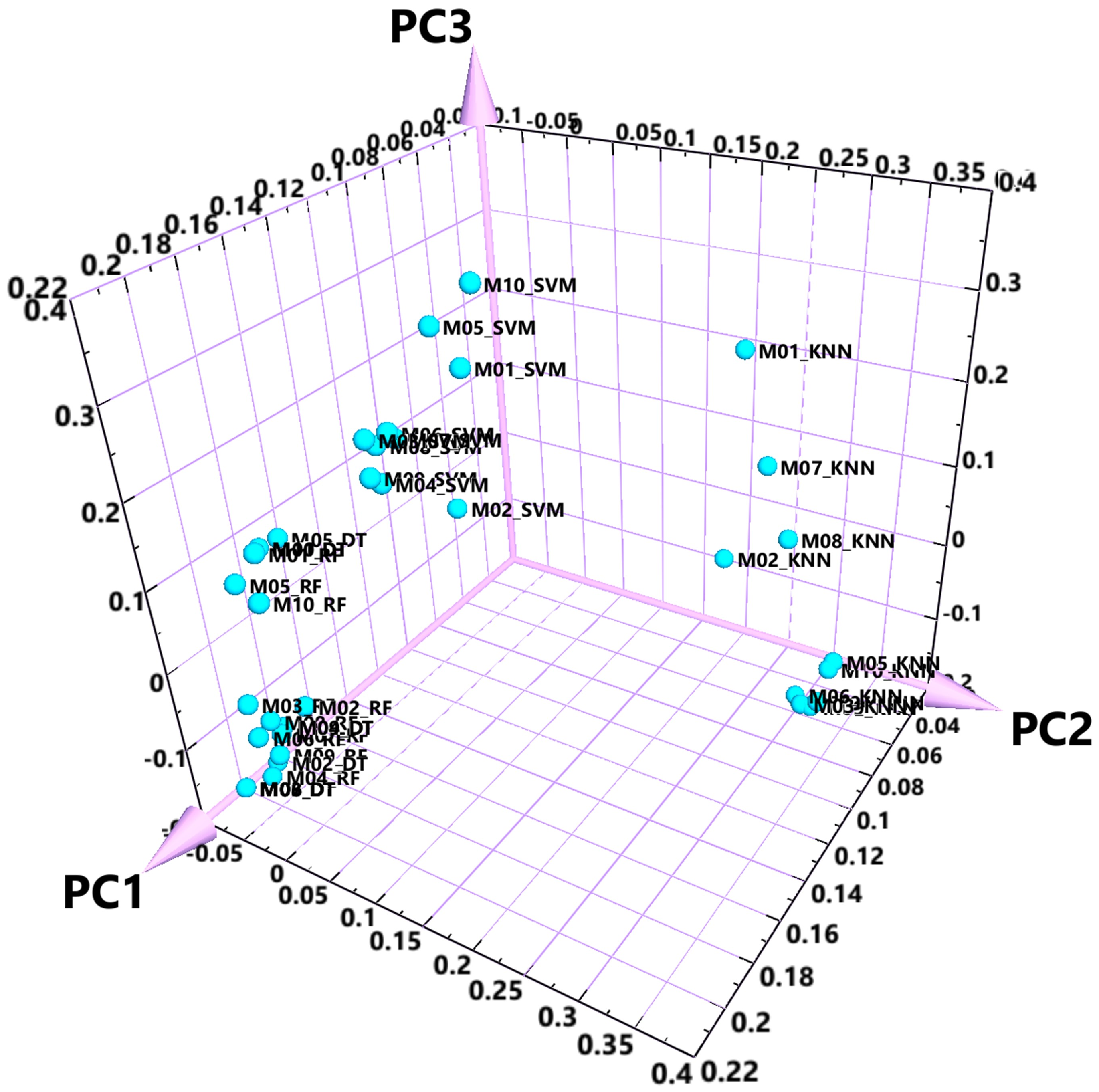


**(c)**
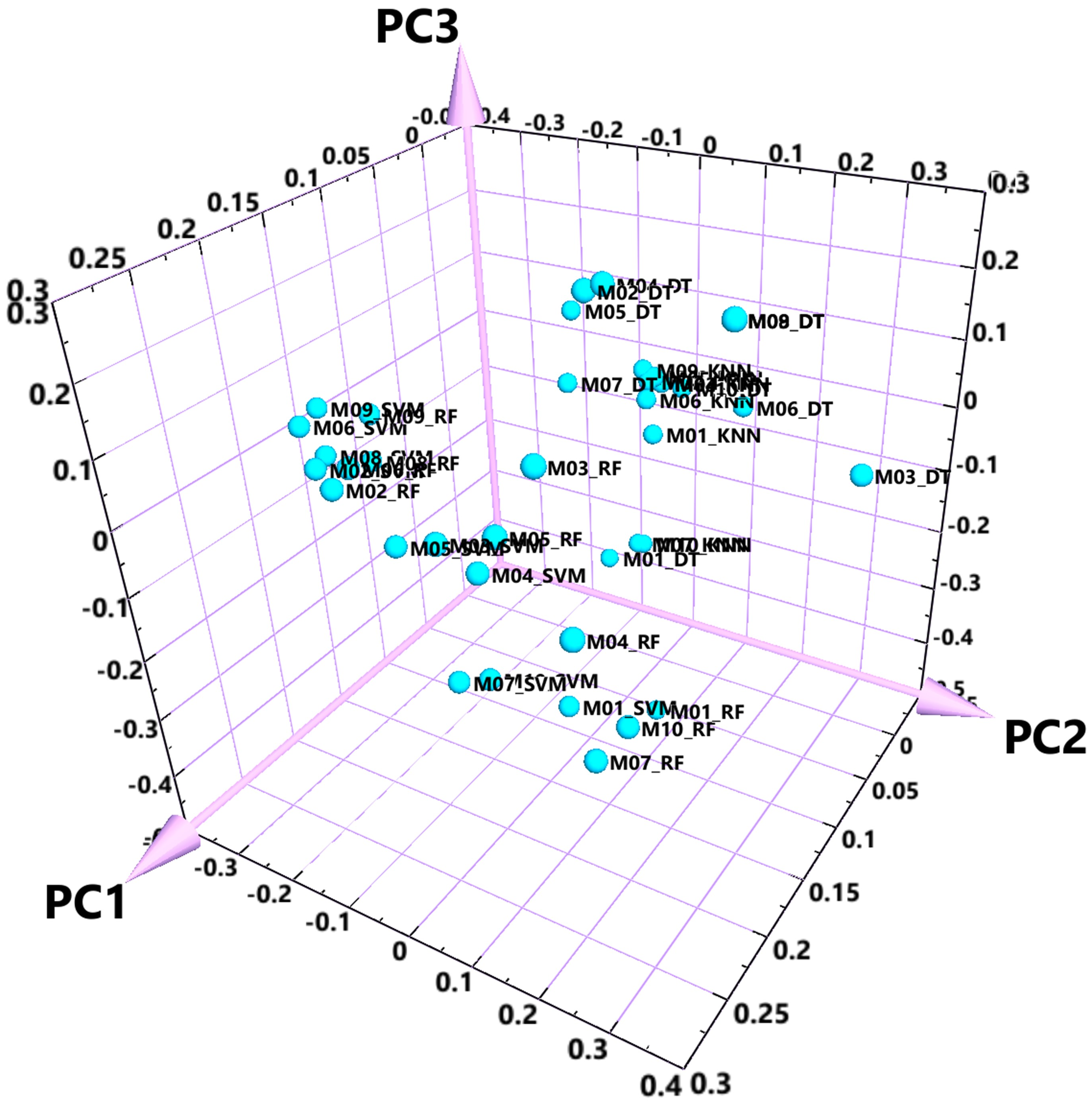


**(d)**


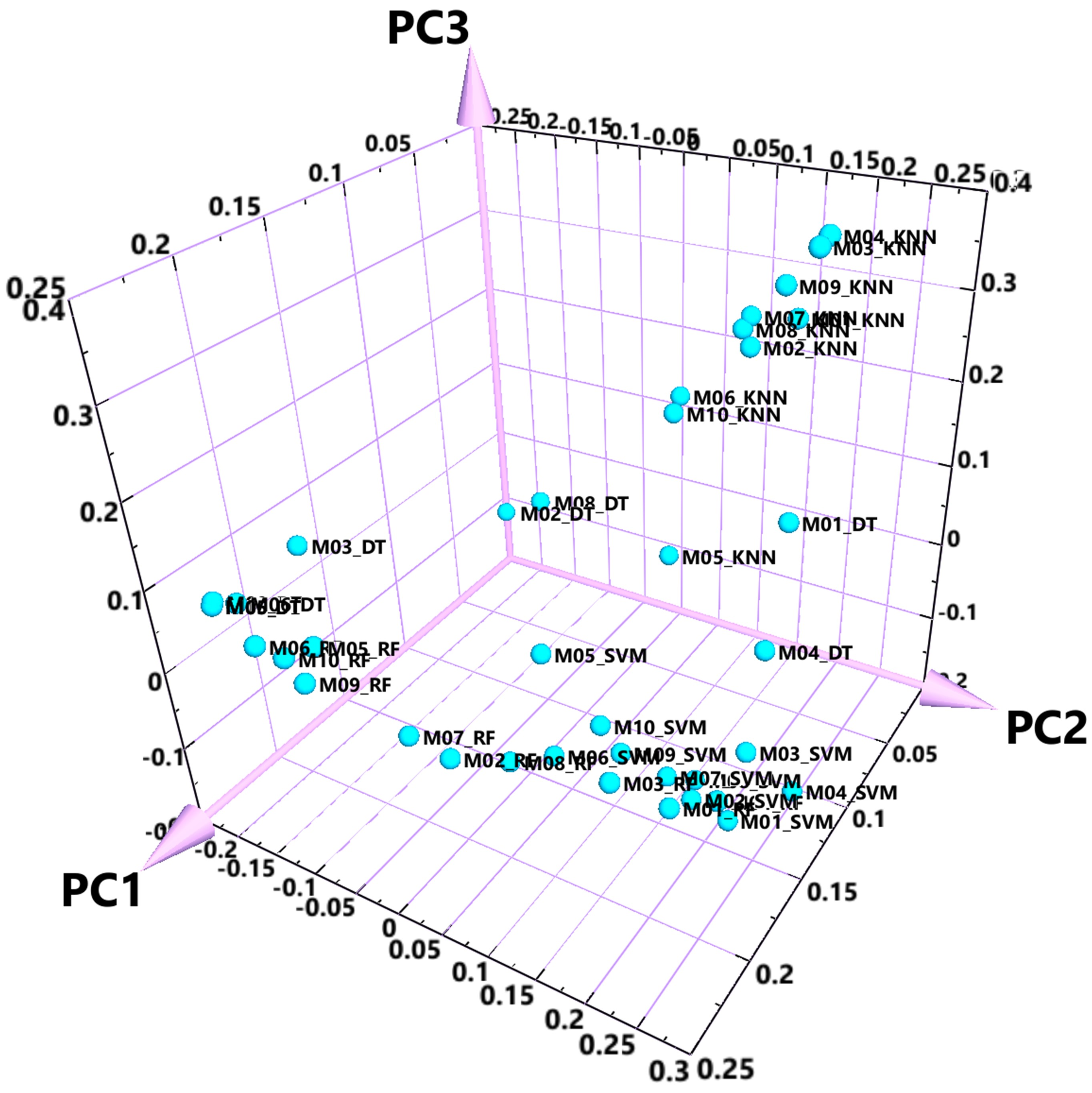


**(e)**


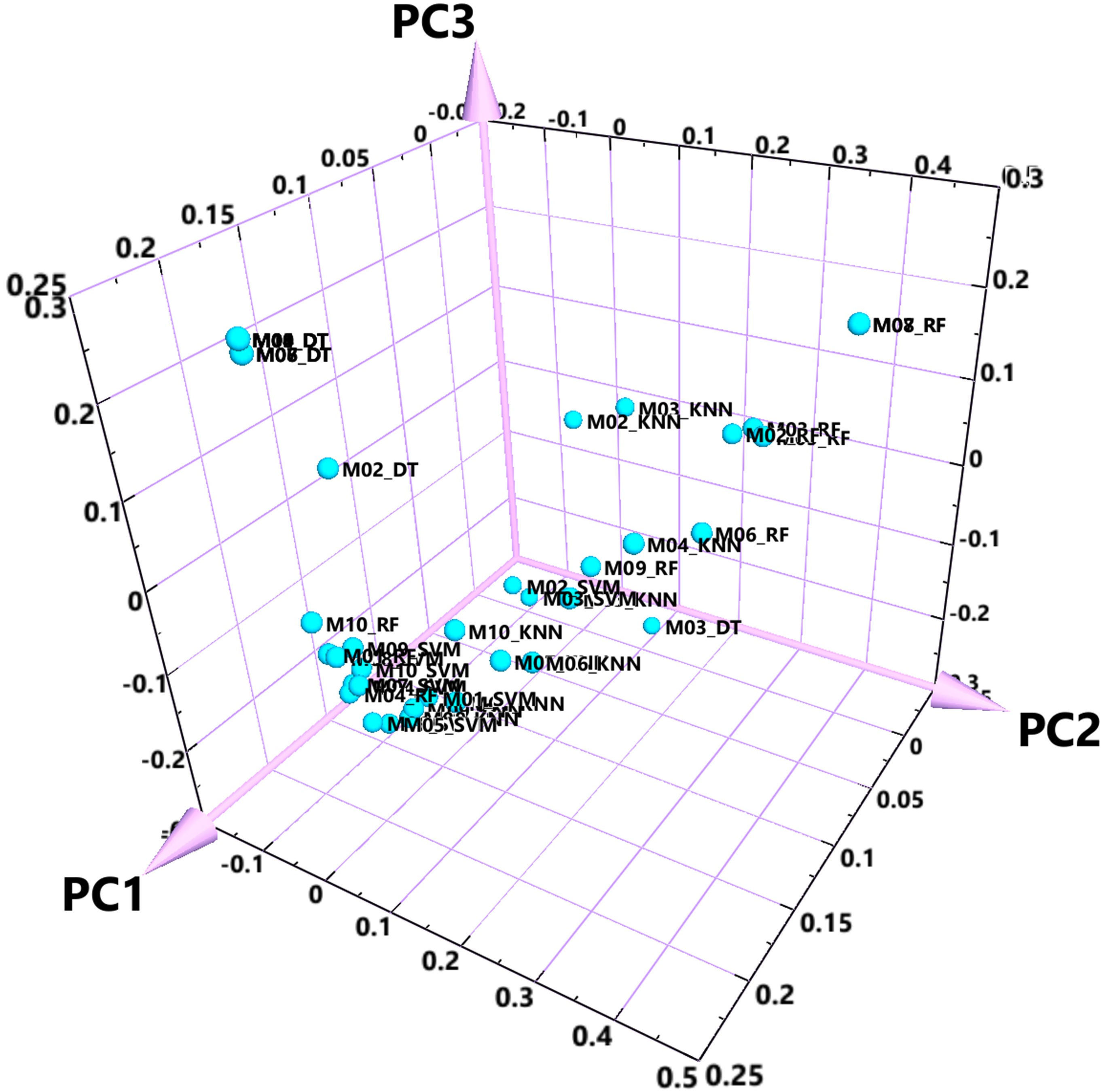


**Figure S8:** The workflow to manipulate the prediction results of CWA and NPS.

**
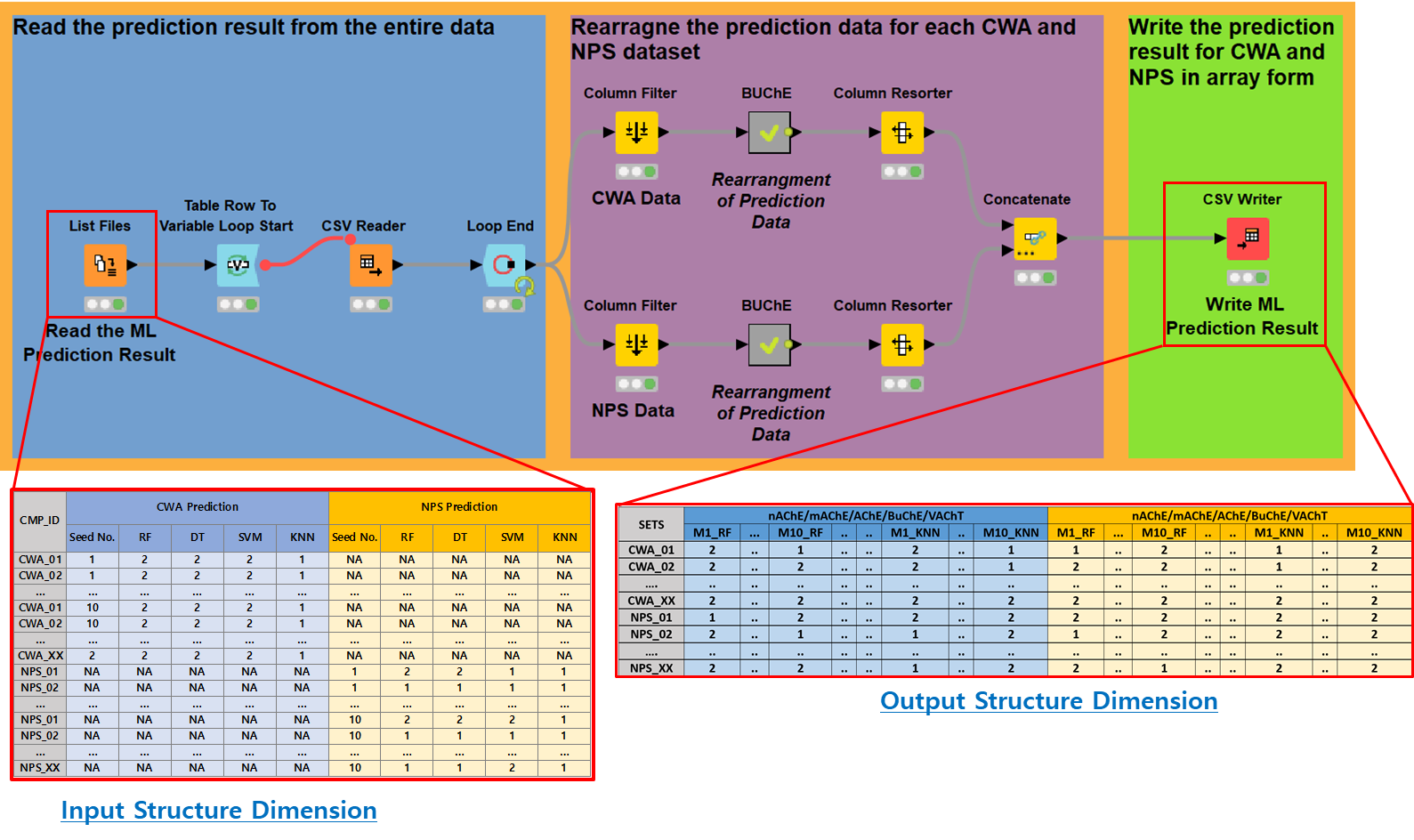
**

Given the input of different target prediction result from machine learning models, this KNIME workflow allows anyone to transform the input data into an array matrix with the dimensions of (4ML * 5targets * 10models = 200*number of compounds).

**Figure S9:** A Typical Convolutional neural network (CNN) model architecture used in this study. The architecture shown here is for CNN model with input shape of (50 x 4).


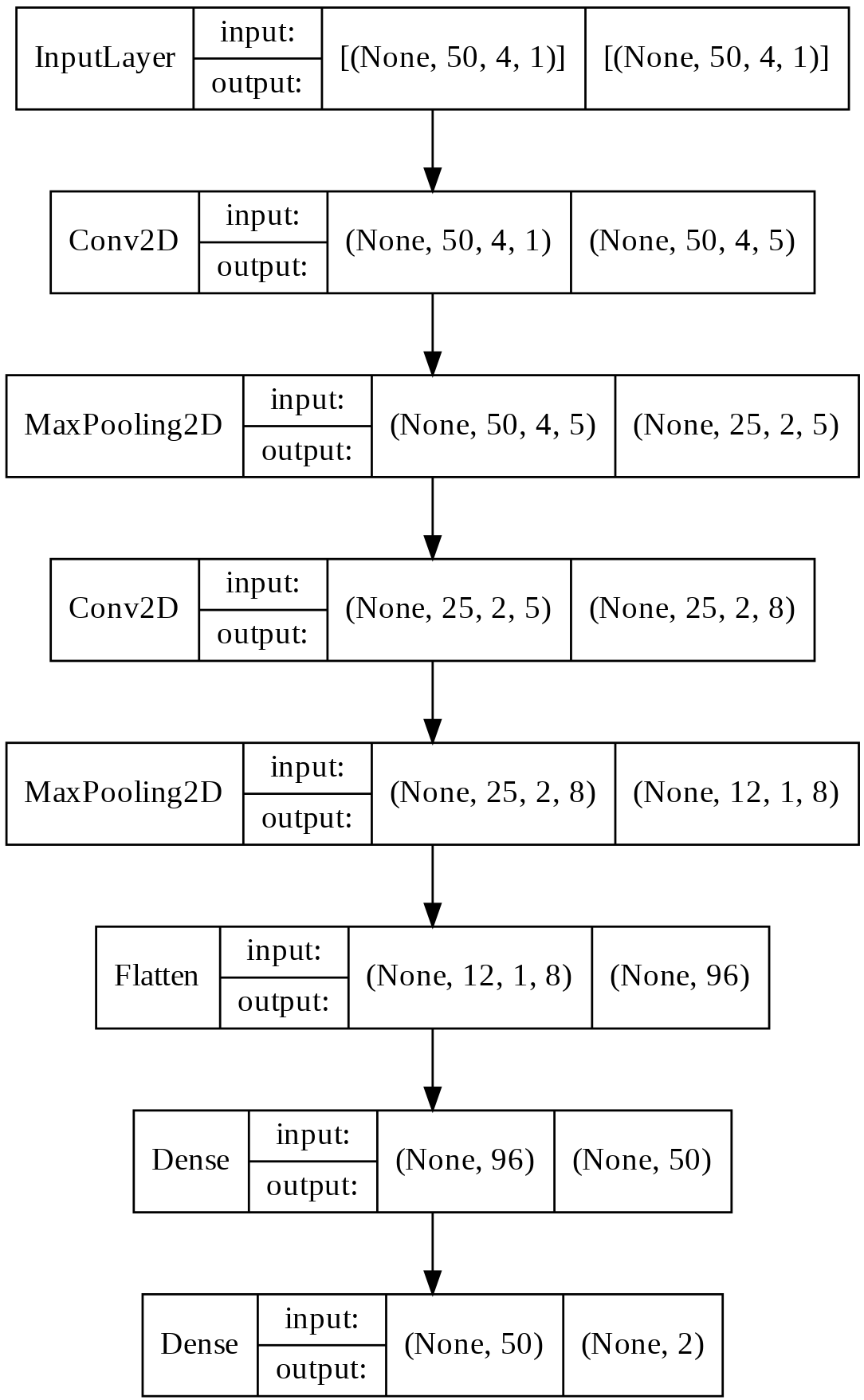


**Table S8:** The statistical performance of (CNN) model under different Array Shapes.

| **CNN Models Shapes** | **Models** | **Train Set** | | **Test Set** | | | | | | | | |
| --- | --- | --- | --- | --- | --- | --- | --- | --- | --- | --- | --- | --- |
|  |  | **ACC** | **Loss** | **ACC** | **Loss** | **MCC** | **CWA** | | | **NPS** | | |
|  |  |  |  |  |  |  | **auROC** | **auPR** | **F1-Score** | **auROC** | **auPR** | **F1-Score** |
| **CNN**  **(50, 4)** | **Model01** | 0.985 | 0.045 | 0.982 | 0.075 | 0.638 | 0.933 | 0.724 | 0.585 | 0.933 | 0.997 | 0.991 |
|  | **Model02** | 0.995 | 0.028 | 0.980 | 0.131 | 0.599 | 0.942 | 0.695 | 0.585 | 0.939 | 0.997 | 0.990 |
|  | **Model03** | 0.998 | 0.014 | 0.977 | 0.138 | 0.581 | 0.925 | 0.688 | 0.593 | 0.936 | 0.997 | 0.988 |
|  | **Model04** | 0.937 | 0.445 | 0.864 | 1.031 | 0.348 | 0.921 | 0.360 | 0.298 | 0.917 | 0.996 | 0.925 |
| **CNN-3D (10, 5, 4)** | **Model01** | 0.989 | 0.047 | 0.979 | 0.117 | 0.567 | 0.928 | 0.639 | 0.545 | 0.927 | 0.996 | 0.989 |
|  | **Model02** | 0.985 | 0.039 | 0.976 | 0.106 | 0.558 | 0.929 | 0.569 | 0.565 | 0.929 | 0.997 | 0.988 |
|  | **Model03** | 0.988 | 0.043 | 0.963 | 0.156 | 0.485 | 0.938 | 0.627 | 0.486 | 0.938 | 0.998 | 0.981 |
|  | **Model04** | 0.943 | 0.413 | 0.883 | 1.017 | 0.392 | 0.920 | 0.474 | 0.340 | 0.922 | 0.996 | 0.936 |
| **CNN-3D Reshaped (5, 10, 4)** | **Model01** | 0.980 | 0.058 | 0.978 | 0.092 | 0.537 | 0.907 | 0.606 | 0.512 | 0.907 | 0.995 | 0.989 |
|  | **Model02** | 0.960 | 0.097 | 0.941 | 0.132 | 0.397 | 0.925 | 0.611 | 0.395 | 0.925 | 0.994 | 0.969 |
|  | **Model03** | 0.988 | 0.063 | 0.952 | 0.262 | 0.470 | 0.957 | 0.696 | 0.452 | 0.953 | 0.998 | 0.975 |
|  | **Model04** | 0.940 | 0.443 | 0.875 | 0.963 | 0.364 | 0.930 | 0.419 | 0.316 | 0.917 | 0.995 | 0.931 |
| **Baseline Model** | **Model01** | 0.982 | - | 0.979 | - | 0.583 | 0.689 | 0.366 | 0.540 | 0.672 | 0.980 | 0.990 |

**Abbreviations: Model01:** Model without SMOTE; **Model02:** Model without SMOTE and without Duplicates; **Model03:** Model with SMOTE; **Model04:** Model with SMOTE and without duplicates; **ACC:** Accuracy; **MCC:** Matthew’s Correlation Coefficient; **auROC:** Area-Under-Receiver Operating Characteristic Curve; **auPR:** Area-Under-Precision-Recall Curve; **CWA:** Chemical Warfare agents**; NPS:** New Psychoactive Substances. **Baseline Model**: The baseline model is built on Multilayer Perceptron (MLP) Classifier. The Best model was based on the data of CNN (50,4) Model01 and was compared with CNN Models.
